# The Chinese herbal formula *Free and Easy Wanderer* ameliorates oxidative stress through KEAP1-NRF2/HO-1 pathway

**DOI:** 10.1038/s41598-017-10443-6

**Published:** 2017-09-14

**Authors:** Chunlan Hong, Jingming Cao, Ching-Fen Wu, Onat Kadioglu, Anja Schüffler, Ulrich Kauhl, Sabine M. Klauck, Till Opatz, Eckhard Thines, Norbert W. Paul, Thomas Efferth

**Affiliations:** 1 Department of Pharmaceutical Biology, Institute of Pharmacy and Biochemistry, Johannes Gutenberg University, Staudinger Weg 5, 55128 Mainz, Germany; 2 Institute of Biotechnology and Drug Research, Kaiserslautern, Germany; 3 Institute of Organic Chemistry, Johannes Gutenberg University, Mainz, Germany; 4 Division of Cancer Genome Research, German Cancer Research Center (DKFZ), German Cancer Consortium (DKTK), National Center for Tumor Diseases (NCT), Heidelberg, Germany; 5 Institute of Biotechnology, Johannes Gutenberg University, Mainz, Germany; 6 Institute of History, Theory and Ethics of Medicine, Johannes Gutenberg University Medical Center, Mainz, Germany

## Abstract

Posttraumatic stress disorder (PTSD) gains a lot of attention due to high prevalence and strong psychological upset, but the etiology remains undefined and effective treatment is quite limited. Growing studies demonstrated the involvement of oxidative stress in various psychiatry diseases, suggesting anti-oxidation therapy might be a strategy for PTSD treatment. *Free and Easy Wanderer* (FAEW) is a poly-herbal drug clinically used in China for hundreds of years in the treatment of psychiatric disorder. We hypothesized that FAEW exerts clinical effects through the activity against oxidative stress with fluoxetine as antidepressant control drug. Our results revealed that FAEW significantly reduced both endogenous and H_2_O_2_-induced exogenous ROS levels in the human glioblastoma T98G and neuroblastoma SH-SY5Y cell lines. Transcriptome-wide microarray analysis indicated NRF2/HO-1 as the common target of FAEW and fluoxetine. Western blotting assay proved that the two drugs promoted NRF2 release from KEAP1 in the cytoplasm and translocation to the nuclei in a KEAP1-dependent manner, the expression of the protein HO-1 increased accordingly, suggesting the participation of KEAP1-NRF2/HO-1 pathway. The chemical constituents of FAEW (*i.e*. paeoniflorin, baicalin) bound to KEAP1 *in silico*, which hence might be the effective substances of FAEW. In conclusion, FAEW counteracted H_2_O_2_-induced oxidative stress through KEAP1-NRF2/HO-1 pathway.

## Introduction

Posttraumatic stress disorder (PTSD) may develop after a person is exposed to a traumatic event, characterized by repeatedly experiencing trauma with disturbing recurring flashbacks, avoidance or numbing of memories of the event, and hyper-arousal^1–4^. It was first recognized as a distinct disease among US-American Vietnam veterans and becomes popular due to high prevalence, strong psychological upset and difficult prediction. The life time prevalence of PTSD in adults is 7.8%, women have a higher risk than men (20.4% vs. 8.2%)^5, 6^. Current treatment options for PTSD are mainly limited to psychotherapy, such as trauma-focused cognitive-behavior therapy (TFCBT), exposure therapy, eye movement desensitization and reprocessing (EMDR)^7^. Sertraline (Zoloft^®^) and paroxetine (Paxil^®^) are the only two drugs approved by the Food and Drug Administration (FDA) for the treatment of PTSD^8–10^. Therefore, novel effective drugs or alternative medicine are urgently needed, for instance, traditional Chinese herbs might be beneficial for the treatment of PTSD.

Oxidative stress, as the main endogenous source of DNA damage, can destroy cellular vital components like DNA, proteins, and lipid by resulting in free radicals from oxygen metabolism as byproducts such as reactive oxygen species (ROS). DNA damage caused by oxidative stress in the nuclei and mitochondria may even block genome replication and transcription, which further result in mutations or genome aberration. A growing body of studies demonstrated involvement of oxidative stress in psychiatric disorder, and oxidative damage in the brain of the patients suffering from major mental illnesses was proved to be one of the major pathological processes^11^. Elevated lipid peroxidation were shown in the patients of generalized anxiety disorder as well as suppressed antioxidant activity in panic disorder^12^. The link between oxidative stress-related genes and stress-related phenotypes has been determined by a GWAS of PTSD^13^. Recently, a novel locus in the oxidative stress-related gene *ALOX12* has also been identified^14^, suggesting the involvement of oxidative stress in PTSD. Antioxidant therapy, thereby, may become a treatment strategy for psychiatric disorder.

*Free and easy wanderer* (FAEW) is a poly-herbal preparation. It has been long used in China for the treatment of depression, premenstrual dysphoric disorder, climacteric syndrome, and Parkinson’s disease. *In vivo* studies revealed that FAEW acted against anxiety and improved cognition levels^15^. Clinical trials confirmed its effect in the treatment of depression^16^. Although FAEW has a good reputation in the history of Chinese medicine and shows great potential in the treatment of PTSD, the cellular and molecular modes of action are still not well understood.

In order to explain the mechanisms of FAEW on mental diseases, we firstly used H_2_O_2_ to induce oxidative stress in the human glioblastoma T98G and human neuroblastoma SH-SY5Y cell lines, and examined the effect of FAEW and fluoxetine on ROS levels. To identify underlying cellular mechanisms, we furthermore performed transcriptome-wide microarray analyses. In the course of these investigations, we verified the role of KEAP1-NRF2 and their downstream gene HO-1. Finally, molecular docking was performed to explore possible phytochemicals of FAEW, which bind to the NRF2-regulator Kelch-like ECH-associated protein 1 (KEAP1).

## Results

### Cytotoxicity of FAEW and fluoxetine

**Figure 1:**
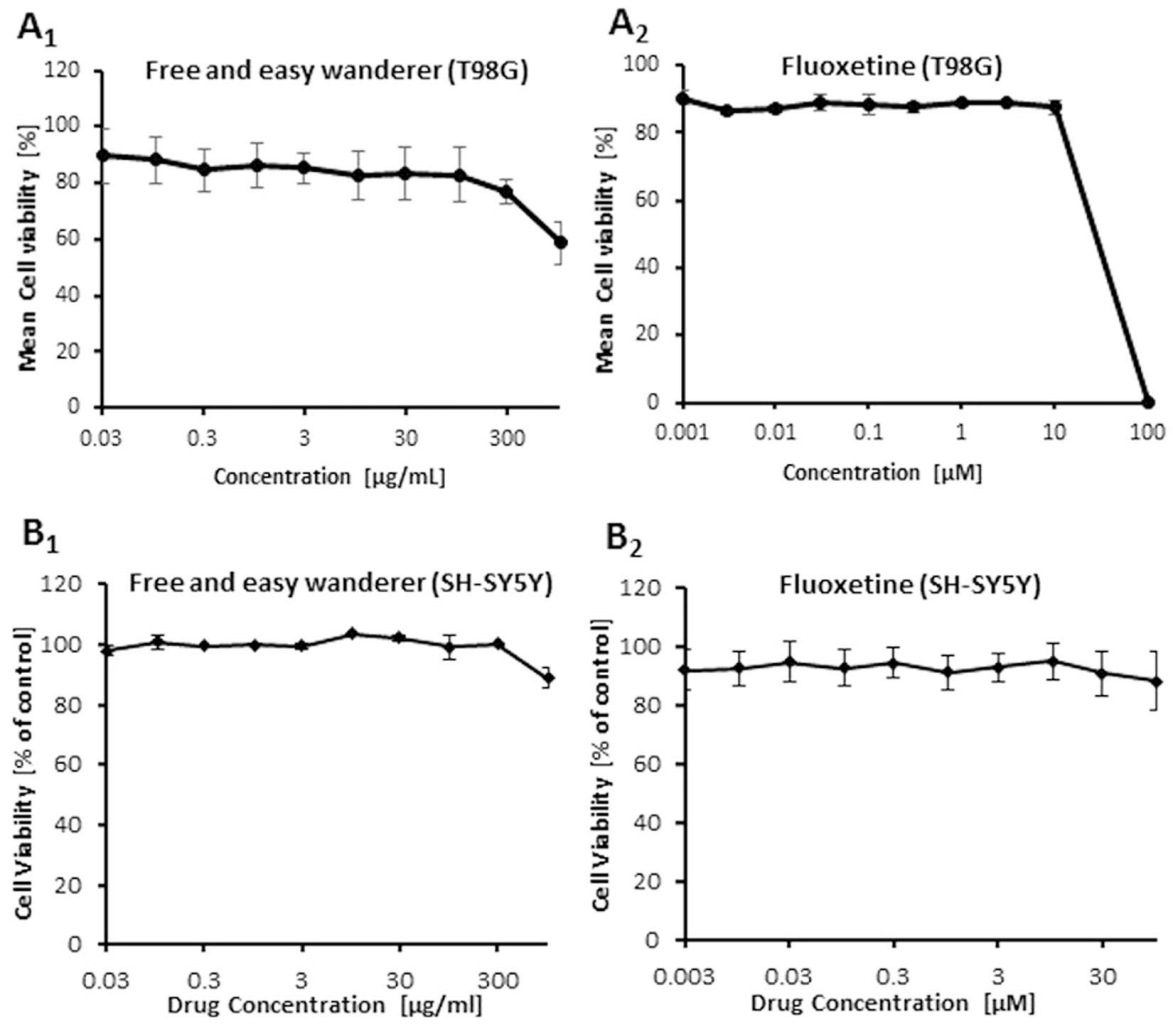
Cytotoxicity of FAEW (1) and fluoxetine (2) determined by the resazurin assay in the human glioblastoma T98G (**A**) and human neuroblastoma SH-SY5Y (**B**) cells. Shown are mean values ± SD of three independent experiments.

As a first step, we performed resazurin assays in human glioblastoma T98G and human neuroblastoma SH-SY5Y cell lines to investigate whether or not FAEW reveals cytotoxic effects. As expected, FAEW was indeed not cytotoxic towards T98G cells at concentrations up to 300 µg/ml (Fig. 1A_1_). For comparison, fluoxetine was non-toxic up to 3.1 µg/ml (10 µM) and inhibited T98G cells at higher concentrations (Fig. 1A_2_). However, both FAEW and fluoxetine have no cytotoxicity towards SH-SY5Y cell, even at higher concentrations (Fig. 1B_1_, B_2_).

### Inhibition of ROS by FAEW and fluoxetine

**Figure 2:**
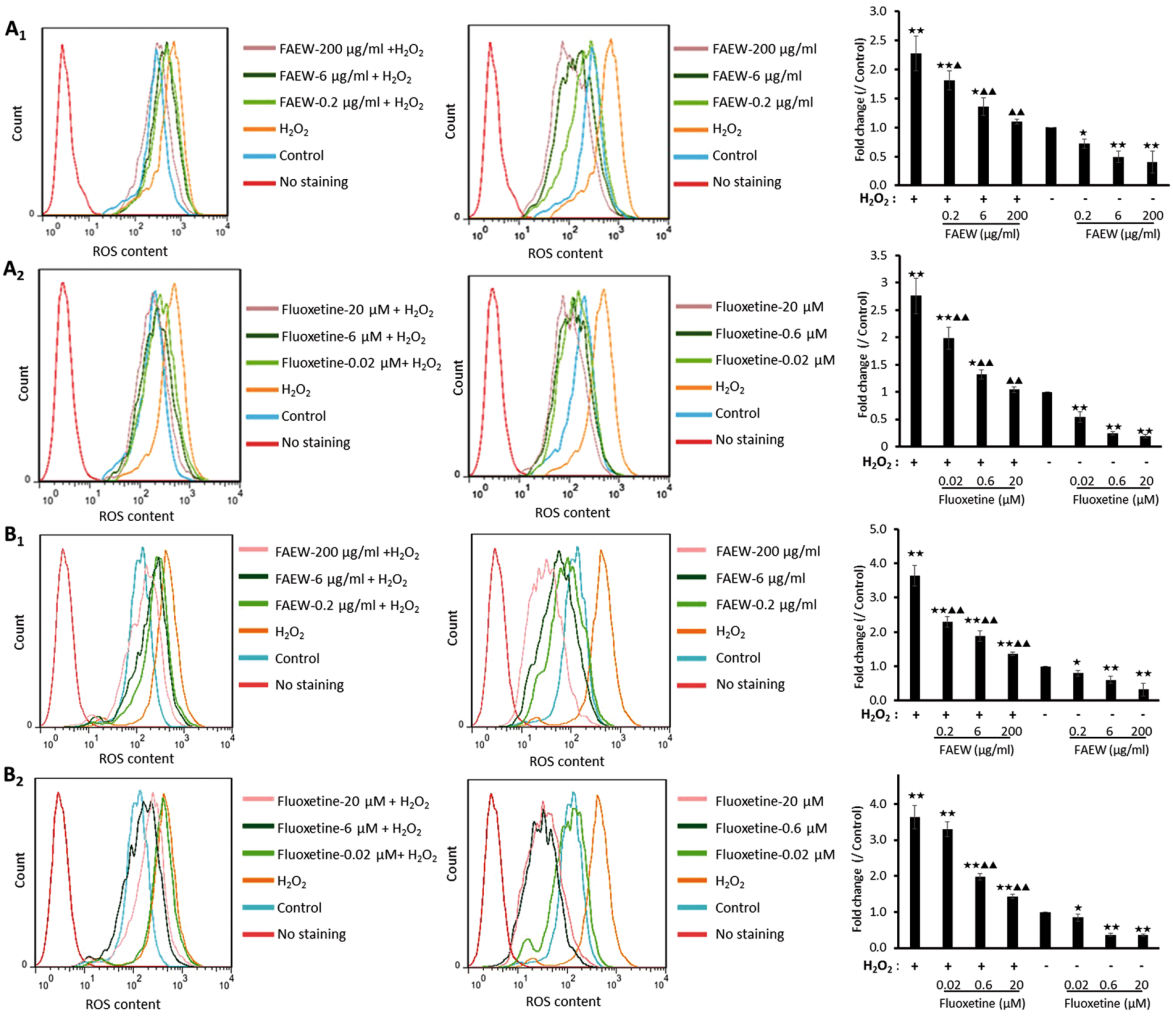
Inhibition of reactive oxygen species by FAEW (1) or fluoxetine (2) in the human glioblastoma T98G (**A**) and human neuroblastoma SH-SY5Y (**B**) cells. Shown are mean values ± SD of three independent experiments. ^★^ *p* < 0.05, ^★★^ *p* < 0.01, compared with control; ^▲^ *p* < 0.05, ^▲▲^ *p* < 0.01, compared with H_2_O_2_.

We selected several non-cytotoxic concentrations of FAEW and fluoxetine, and performed flow cytometry analysis to investigate ROS levels. Firstly, we used hydrogen peroxide (H_2_O_2_) to induce oxidative stress *in vitro*. As shown in Fig. 2A_1_ and B_1_, FAEW strongly reduced the levels of ROS induced by H_2_O_2_ and the levels of ROS in untreated T98G and SH-SY5Y cells, indicating that FAEW diminished exogenous H_2_O_2_-induced ROS and also protected against endogenous ROS. Fluoxetine showed the same effect as FAEW as shown in Fig. 2A_2_ and B_2_.

### Gene expression profiling caused by FAEW and fluoxetine

Gene expression profiling was performed to obtain deeper insight into the mechanisms of FAEW and fluoxetine against oxidative stress. Therefore, total RNA was extracted to perform transcriptome-wide microarray analyses, after human glioblastoma T98G cells were treated with FAEW or fluoxetine for 48 h, and oxidative stress was induced with H_2_O_2_ for 6 h.

**Figure 3:**
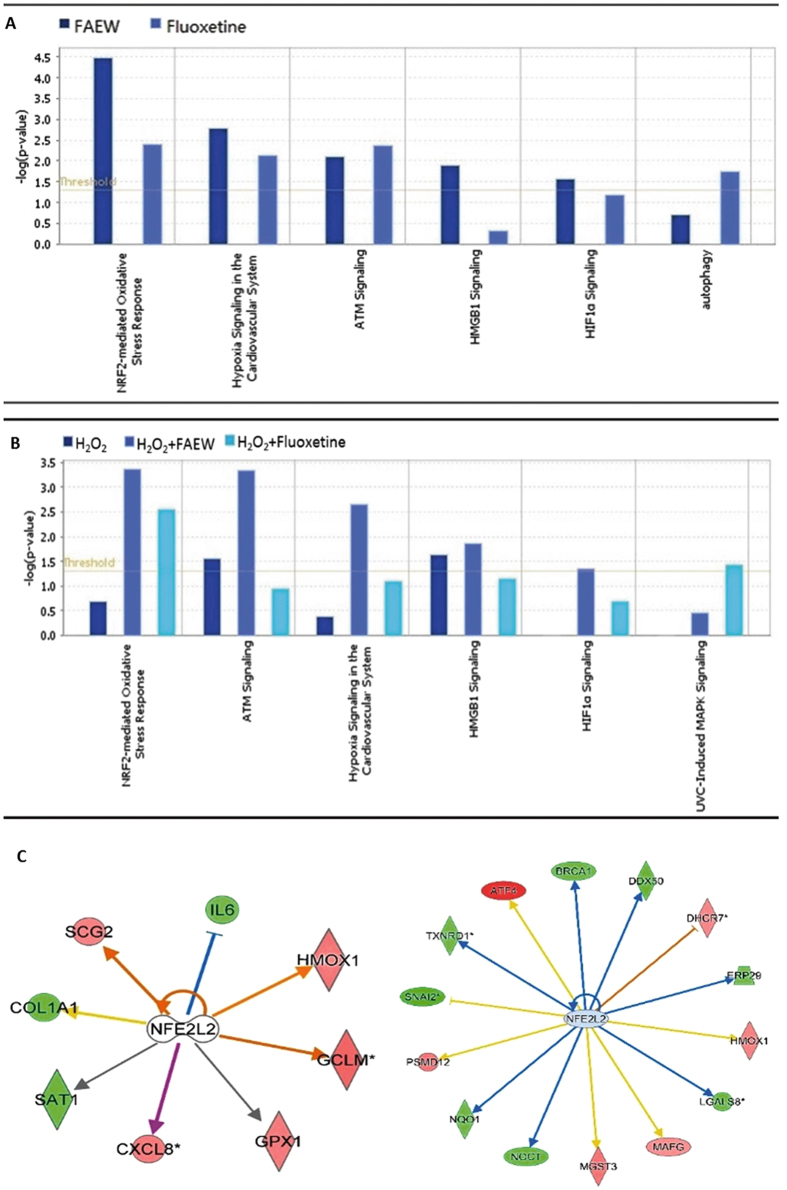
Gene expression profiling of T98G cells upon treatment of FAEW or fluoxetine. (**A** and **B**) Pathway analyses: Top cellular pathways affected by FAEW and fluoxetine examined by mRNA microarray hybridization. (**A**) Shows the comparison between FAEW and fluoxetine. (**B**) Shows the comparison between H_2_O_2_, H_2_O_2_ and FAEW, H_2_O_2_ and fluoxetine. *P*-values were calculated using right-tailed Fisher’s exact test. (**C**) Deregulated genes under the influence of the common upstream regulator NFE2L2 (NRF2) inhibited by FAEW (left) and fluoxetine (right).

All data obtained by microarray analyses were subjected to pathway analysis. The deregulated genes were correlated with several molecular and cellular functions and pathways. As shown in Fig. 3A and B, NRF2-mediated oxidative stress was the top-ranked pathway upon FAEW treatment. Furthermore, an upstream regulator analysis was performed with IPA to identify transcriptional regulators, kinases, or enzymes that may be responsible for gene expression changes in T98G cells after treatment. Table 1 shows the upstream transcriptional factors predicted by IPA to be affected by FAEW or fluoxetine. Remarkably, NFE2L2 (alias NRF2) (underlined) was found to be a commonly activated transcription regulator by both FAEW and fluoxetine, implying that NRF2-mediated stress response may be involved with the mechanisms of the two drugs. Table 2 displays the targeted genes downstream of NRF2 upon different treatment models. Most interestingly, *HMOX1* (alias *HO-1*) was commonly targeted by FAEW and fluoxetine with or without oxidative stress. Six deregulated downstream genes of NRF2, including *HMOX1* were quantified by real-time RT-PCR to technically validate the microarray results. KEAP1 was identified as a cytoplasmic NRF2-interacting protein that negatively regulates NRF2 activity, but recent studies revealed that alternative mechanisms of NRF2 activation that do not rely on KEAP1^17^. Therefore, we also investigated the transcriptional levels of NRF2 and KEAP1. The correlation coefficients (R-values) between mRNA expression values determined by microarray hybridization and real-time RT-PCR were in the range of 0.83 to 0.97 for each compound (Pearson correlation test), indicating a high degree of concordance between the data obtained from the two different methods (Table 3). The levels of KEAP1 and NRF2 were not affected, which excluded the transcription regulation and autoregulation, and suggested the possibility of participation of KEAP1-NRF2 protein-protein interaction. Figure 3C shows the deregulated genes controlled by NRF2 upon treatment by FAEW and fluoxetine, respectively.

**Table 1:** Most pronounced upstream transcription factors for deregulated genes upon different treatments.

Comparison with control					Comparison with H_2_O_2_	
FAEW	Fluoxetine	H_2_O_2_	H_2_O_2_ + FAEW	H_2_O_2_ + Fluoxetine	H_2_O_2_ + FAEW	H_2_O_2_ + Fluoxetine
RBPJ	SOX2	KDM5B	NUPR1	TP53	JUN	***NFE2L2***
HMGA1	EGR2	TP53	KDM5B	FOXO1	RELA	PDX1
HLX	ATF4	***NFE2L2***	RELA	TCF7L2	FOXO1	TCF3
CEBPD	TCF3	FOXM1	JUN	NEUROG1	CREB1	HMGA1
***NFE2L2***	GFI1	MYC	CREB1	TFEB	DDIT3	MED1
CTNNB1	SPI1	TBX2	***NFE2L2***	IRF4	NUPR1	MYC
CEBPA	SMAD4	CCND1	REL	RELA	ECSIT	FOXM1
SOX2	HIF1A	TAL1	PPRC1	SRF	JUNB	ERG
EGR1	GATA1	ATF4	RELB	CREBBP	REL	ATF6
RELB	SMARCB1	ATF6	TP53	SP1	***NFE2L2***	RUNX2
JUNB	E2F3	REL	ECSIT	XBP1	STAT3	NUPR1
EBF1	JUN	MED1	SPI1	NUPR1	ATF2	FOS
NKX2-3	MTPN	MITF	WT1	NFKBIA	SOX2	BRCA1
YY1	MDM2	NUPR1	ATF4	***NFE2L2***	FOXL2	SMARCE1
MITF	RUNX2	CEBPB	TP63	NOTCH1	CEBPB	STAT5B
ETS2	KLF4	XBP1	RUNX1	CREB1	RBPJ	SREBF1
GLI1	NKX2-3	BRCA1	NCOA3	VDR	FOXO3	CTNNB1
SRF	IRF3	SRF	TBX2	MEF2C	HIF1A	TFEB
STAT3	HIC1	E2F1	YY1	CTNNB1	TP53	SOX2
SPDEF	TFEB	GPS2	FLI1	BRCA1	CREBBP	TP63
CCND1	MYC	IFI16	CARM1	JUN	NFKB1	NKX2-3
EP300	HMGA1	FOXO1	TFAP2C	HOXA10	RB1	SIRT1
TCF7L2	STAT1	FOS	MEF2D	TAL1	EP300	FOXO4
HDAC6	RB1	IRF3	POU2F1	FOS	NKX2-3	TP73
DACH1	E2F1	STAT3	GATA3	HIC1	EZH2	NFKBIA
FOSL1	NRF1	RB1	IRF6	ELK1	USF1	DDIT3
TP53	BRCA1	CREB1	ATF2	IKZF1	CDKN2A	FOXO3
SMARCA4	TP63	HIF1A	CTNNB1	IFI16	CEBPA	HIF1A
BRCA1	ETS2	HIC1	FOXL2	FOXO3	TP73	CREB1
CITED2	***NFE2L2***	EGR1	MITF	GLI1	SIRT1	GLI1

**Table 2:** Target genes in the corresponding dataset regulated by NRF2.

Comparison	Treatment	Activation z-score	P-value of overlap	Target molecules in dataset
Compared with control	FAEW	1.790	1.49E-02	*CXCL8*, *GCLM*, ***HMOX1***, *GPX1*, *IL6*, *SAT1*, *SCG2*, *COL1A1*
	Fluoxetine	1.000	3.64E-04	*ATF4*, *BRCA1*, *DDX50*, *DHCR7*, *ERP29*, ***HMOX1***, *LGALS8*, *MAFG*, *MGST3*, *NOCT*, *NQO1*, *PSMD12*, *SNAI2*, *TXNRD1*
	H_2_O_2_	2.390	7.08E-02	*ATF4*, *CXCL8*, ***HMOX1***, *PSAT1*, *RRS1*, *VCAM1*
	H_2_O_2_ + FAEW	2.674	3.04E-03	*AHR*, *ATF4*, *BNIP3*, *CREG1*, *CXCL8*, *GCLM*, *GPX1*, ***HMOX1***, *IL6*, *MAFF*, *MGST1*, *NQO1*, *PAFAH1B1*, *RRS1*, *S100P*, *SLC6A9*, *SLC7A11*, *SOD2*, *TXNRD1*, *VCAM1*
	H_2_O_2_ + Fluoxetine	1.685	2.58E-04	*ALDH3A1*, *CREG1*, *DAD1*, *GCLM*, *HERPUD1*, *IL6*, *MGST1*, *MGST3*, *SCG2*, *SRXN1*, ***HMOX1***
Compared with H_2_O_2_	H_2_O_2_ + FAEW	1.837	4.25E-03	*CXCL8*, ***HMOX1***, *IL6*, *MAFF*, *SOD2*
	H_2_O_2_ + Fluoxetine	2.250	8.91E-02	*CTSD*, *DDIT3*, *DHCR7*, *GCLM*, *IL6*, *SRXN1*

**Table 3:** Comparison of mRNA expressions (fold change) obtained by microarray gene expression profiling and real-time RT-PCR for selected genes.

Genes	FAEW		Fuoxetine		H_2_O_2_		H_2_O_2_ + FAEW		H_2_O_2_ + Fluoxetine	
	RT-PCR	Microarray	RT-PCR	Microarray	RT-PCR	Microarray	RT-PCR	Microarray	RT-PCR	Microarray
*HMOX1*	2.36	1.72	3.74	1.75	1.91	1.69	5.23	3.46	1.81	1.45
*MGST1*	1.42	1.25	−1.08	−1.15	1.44	1.25	1.50	1.61	1.37	1.75
*TXNRD1*	1.88	1.26	2.87	2.14	2.31	1.45	2.68	1.51	1.75	1.44
*HERPUD1*	1.47	1.28	1.33	1.11	1.68	1.44	2.02	1.46	1.59	1.46
*KEAP1*	1.19	1.14	1.24	1.15	1.16	1.02	2.23	1.40	1.44	1.32
*GCLM*	1.99	1.51	2.42	1.48	1.30	1.12	1.48	1.60	1.97	1.65
*NFE2L2*	−1.20	−1.15	1.22	1.08	1.21	1.13	−1.15	−1.04	−1.06	−1.12
*ATF4*	1.05	1.22	2.38	1.80	1.79	1.52	2.25	1.84	1.45	1.22
R-value	0.97		0.92		0.83		0.96		0.97	

### Inhibition of KEAP1-NRF2 interaction and activity by FAEW and fluoxetine

**Figure 4:**
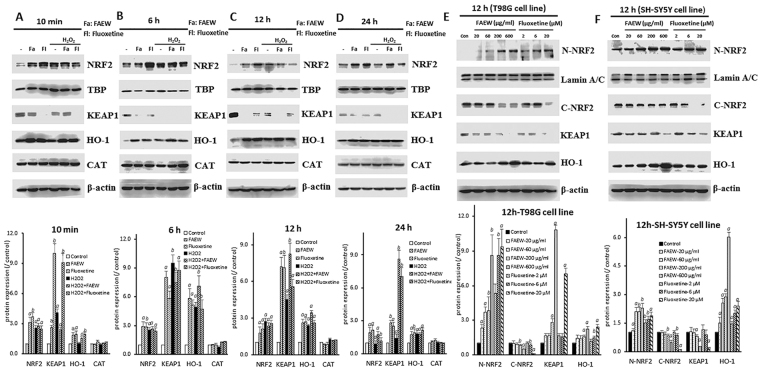
NRF2, HO-1, CAT and KEAP1 protein expression affected by FAEW or fluoxetine in human glioblastoma T98G cells and human neuroblastoma SH-SY5Y cells. (**A**–**D**) Show NRF2, HO-1, CAT and KEAP1 protein expression affected by FAEW or fluoxetine in human glioblastoma T98G cells with different induction times for H_2_O_2_ (**A**), 10 min; (**B**), 6 h; (**C**), 12 h; (**D**), 24 h, respectively. TBP was used as loading control for nuclear proteins and β-actin was used as loading control for total protein and cytoplasmic proteins. (**E** and **F**) show cytoplasmic and nuclear NRF2, cytoplasmic KEAP1 and total HO-1 protein expression in human glioblastoma T98G (**E**) and human neuroblastoma SH-SY5Y (**F**) cells with different concentrations of FAEW and fluoxetine for 12 h. Lamin A/C was used as loading control for nuclear protein, β-actin was used as loading control for total protein and cytoplasmic protein. All the blots were cropped according to their locations in the membrane in order to fit the size, see the original figures in the supplementary information. Shown are mean values ± SD of three independent experiments. ^*a*^ *p* < 0.01, ^*b*^ *p* < 0.05.

To further investigate the protective mechanism of FAEW against oxidative stress, we performed western blotting with NRF2 pathway-related proteins to clarify the participation of the NRF2-HO-1 pathway. As shown in Fig. 4A–D, FAEW and fluoxetine both promoted nuclear NRF2 translocation. The expression levels of NRF2 in the nucleus significantly increased with or without stressed conditions induced by H_2_O_2_. Meanwhile, the levels of total HO-1 also significantly increased, which confirmed the prediction by microarray analysis. H_2_O_2_ also triggered NRF2 translocation at short times, *i.e*. 10 min and 6 h. The effects diminished with longer times of H_2_O_2_-induced stress, *i.e*. 12 to 24 h. The HO-1 levels significantly increased after stress induction for 6 h, the trend lasted for 24 h. While, there is no significant change for the protein expression of catalase (CAT) upon the different treatment. Interestingly, in parallel with the translocation of NRF2 from the cytoplasm, cytosolic KEAP1 levels decreased, indicating a correlation between KEAP1 and NRF2. Figure 4E and F display the dose-response relation of nuclear NRF2, cytoplasmic NRF2, cytoplasmic KEAP1 and HO-1 upon treatment with FAEW or fluoxetine among human glioblastoma T98G and human neuroblastoma SH-SY5Y cell lines, respectively.

### Structures of 10 phytochemicals from FAEW

Ten compounds were isolated from the herb mixture of FAEW, and their structures were identified by HPLC-NMR and MS, as shown in supplementary information.

### *In silico* molecular docking of compounds from FAEW to Keap1

**Figure 5:**
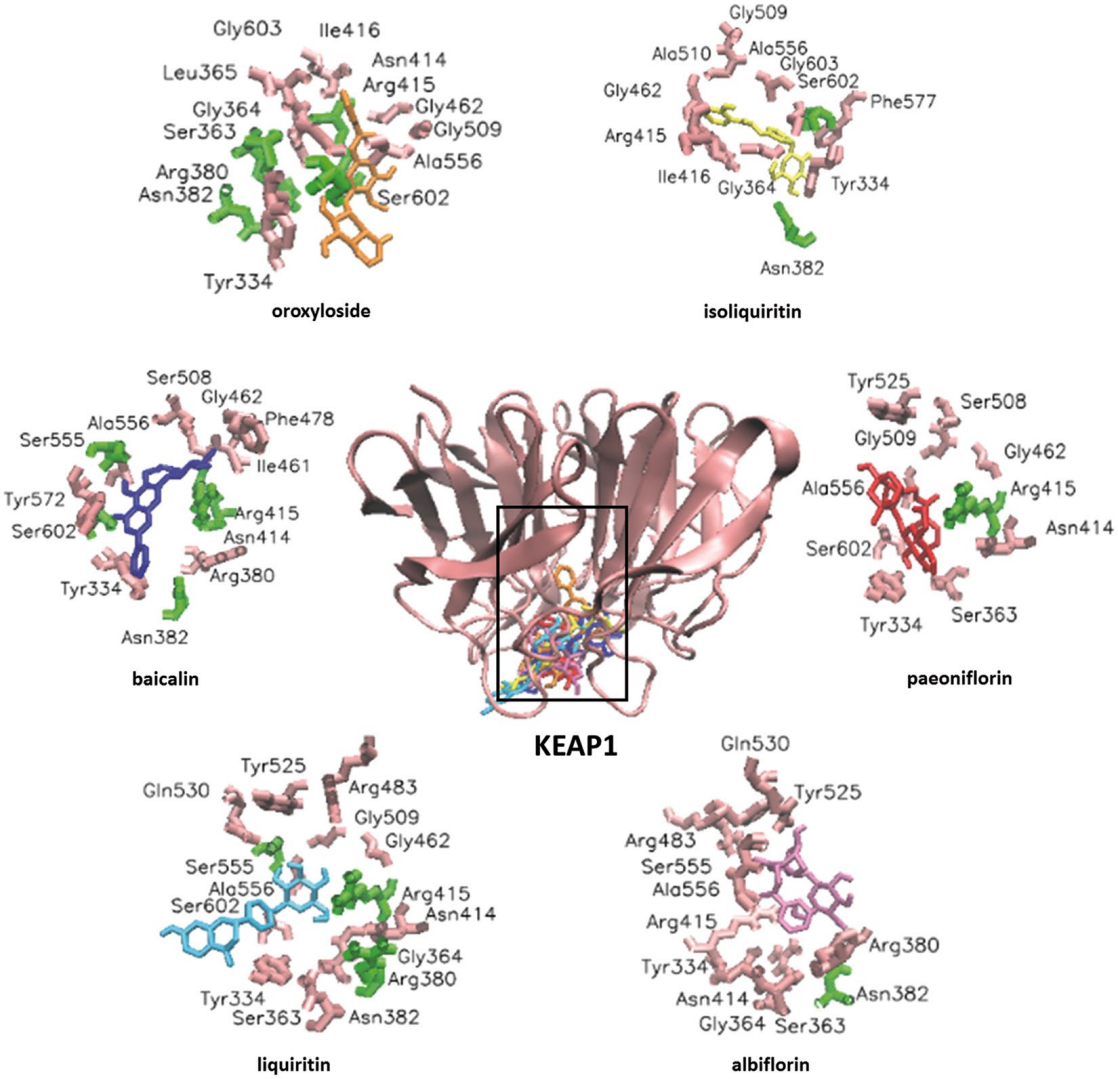
Visualization of molecular docking of chemical compounds isolated from FAEW binding to KEAP1.

Initially, we performed molecular dockings to predict binding energies of 10 compounds from FAEW (supplementary information), the antidepressant control drug fluoxetine and another control drug, IQK, which is a KEAP1-NRF2 interaction inhibitor. Except for isoliquiritin apioside and pentagalloyl-β-D-glucose, the other compounds were predicted to exhibit higher binding affinities than fluoxetine (−5.01 kcal/mol for fluoxetine (S)), especially baicalin (−7.45 kcal/mol), oroxyloside (−7.89 kcal/mol) and liquiritin (−6.86 kcal/mol). Figure 5 shows the predicted binding sites of the compounds to KEAP1.

### Anti-oxidative effect of the compounds isolated from FAEW extract

Among the compounds isolated from FAEW, paeoniflorin, albiflorin, baicalin, and isoliquiritin have been reported against oxidative stress *in vitro*, they are active against apoptosis, neurotoxicity and oxidative injury, *In vivo* experiments revealed their effect in quite many diseases, such as chronic obstructive pulmonary disease, diabetics and colitis. In addition, liquiritin, isoliquiritin apioside are active against oxidative stress-induced genotoxicity, as shown in Table 4.

**Table 4:** Literature review of the effect of chemical constituents of FAEW against oxidative stress.

Compounds	*In vitro*	*In vivo*
Paeonifloirin	Neurotoxicity^43^; apoptosis^44–46^; oxidative injury^47^; cell damage^48^; osteoblast cytotoxicity^49^	Cholestasis^50^, chronic obstructive pulmonary disease^4^
Albiflorin	Neurotoxicity^51, 52^, oxidative stress^52^	Diabetics^53^
Baicalin	Oxidative injury^54, 55^; apoptosis^56^	Colitis^57^; *Haemophilus parasuis* infection^58^; vascular inflammation^59^; neurotoxicity^60^; skin fibroblast^61^
Isoliquiritin	Neurotoxicity^62^	Oxidative stress-induced genotoxicity^63^
Isoliquiritin apioside		Oxidative stress-induced genotoxicity^63^
Liquiritin		Myocardial fibrosis^64^, depression^65^, lung epithelial cell injury^66^, cognitive deficits^67^, endothelial dysfunction^68^

## Discussion

Oxidative stress occurs if molecular defense systems fail to counteract oxidation caused by endogenous processes such as the mitochondrial breakdown of glucose for energy supply or by exogenous xenobiotic chemicals, air pollution and diet. It reflects an imbalance of a biological system’s ability to detoxify reactive intermediates or repair resulting damage. Due to the essential role for physical well-being, longevity and survival, oxidative stress is involved in a variety of diseases, including diabetes, cardiovascular illnesses and neurodegenerative conditions. ROS are byproducts of aerobic metabolism, including the superoxide anion (O_2_^−^), hydrogen peroxide (H_2_O_2_), and hydroxyl radicals (OH^−^). All of them have inherent chemical properties that confer reactivity to different biological targets. Rats under stress conditions exhibited more anxiety-like behavior and elevated ROS levels than corresponding control animals, and *vice versa*, induction of ROS generation resulted in anxiety-like behavior^18^. These observations suggest a causative link between ROS and mental diseases. In our studies, we therefore, employed exogenous H_2_O_2_ for ROS induction in human glioblastoma T98G and human neuroblastoma SH-SY5Y cell lines as the cellular stress model to investigate the effect of FAEW and fluoxetine against oxidative stress. Our results indicated that ROS levels significantly increased upon H_2_O_2_ treatment. Both FAEW and fluoxetine effectively diminished ROS generated by H_2_O_2_. Furthermore, they also significantly reduced the levels of endogenous ROS in untreated cells, suggesting their capability against oxidative stress by removing both endogenous and exogenous ROS.

The multifunctional regulator nuclear factor erythroid 2-related 2 (NFE2L2 or NRF2) is a basic leucine zipper (bZIP) transcription factor that regulates the expression of antioxidant proteins to protect against oxidative damage^19^. NRF2 modulates the expression of well-known antioxidant enzymes, such as HO-1 and glutathione S-transferases (GST), but also a large number of genes that seemingly control disparate processes such as immune and inflammatory responses, tissue remodeling and fibrosis and even cognitive dysfunction and addictive behavior^20, 21^. To this end, NRF2 is referred to as “master regulator” of antioxidant response and is involved in many diseases. Recent studies indicated that NRF2 may play an essential role in the central nervous system. Nrf2-dependent persistent oxidative stress resulted in stress-induced vulnerability to depression in rats^22^. The Kelch-like ECH associated protein 1 (KEAP1)-NRF2 signaling is involved in depression in a mouse model^23^, implying that interruption the KEAP1-NRF2 protein-protein interaction might be a potential therapeutic approach against depression. NRF2 is composed of six functional domains known as NRF2-ECH homologies (Neh) and designated as Neh1-6, respectively^24^. NRF2 is differently regulated in various situations. Under normal physiological conditions, NRF2 is sequestered in the cytosol and maintained at a low level through KEAP1-dependent ubiquitination and proteasome degradation. In the presence of oxidative stress, such as ROS or electrophilic chemicals, the cysteine residues of KEAP1 are covalently modified. These chemical modifications result in conformational changes in KEAP1 that relieve NRF2 from KEAP1-directed degradation. NRF2 translocates to the nucleus and activates ARE-dependent gene expression of a series of anti-oxidative and cytoprotective proteins, such as HO-1^25, 26^. In our studies, the nuclear levels of NRF2 increased upon the induction by H_2_O_2_. In parallel, cytoplasmic KEAP1 levels decreased accordingly, which confirmed the interaction of KEAP1 and NRF2 under stress situations shown in previous studies^27^.

KEAP1 is composed of three functional domains: a bric-a-brac (BTB) domain, an intervening region (IVR), and a Kelch domain (also named DGR domain). KEAP1 forms a homodimer and each dimer binds one molecule of NRF2 via its two Kelch domains, with one weak affinity binding site (DLG motif) and one high affinity binding site (ETGF motif), called “hinge-and-latch”. The KEAP1-NRF2 complex is linked to a functional E3 ubiquitin ligase complex (RBX1) via an adaptor protein, Cullin3. Apart from the general model of KEAP1-CUL3 E3 ligase-mediated NRF2 ubiquitination by targeting KEAP1 cysteine residues, the three-dimensional structure of the KEAP1 Kelch domain, which is responsible for the interaction with NRF2, and the binding cavity of KEAP1 were determined by several groups in mice and humans using X-ray crystallography^28–31^. Subsequently, a variety of KEAP1-NRF2 protein-protein interaction inhibitors were discovered, and the disruption of KEAP1-NRF2 protein-protein interactions became a novel approach for drug discovery of antioxidant agents^32^. Compared with the H_2_O_2_-induced dissociation of NRF2 from KEAP1, both FAEW and fluoxetine alone resulted in the translocation of NRF2 from cytoplasm to the nucleus, as the levels of cytoplasmic NRF2 decreased with the occurrence of the elevation of nuclear NRF2 levels. Besides, the level of nuclear NRF2 increased in a KEAP1-dependent manner, and the antioxidant response element HO-1 significantly elevated at transcriptional and translational levels upon treatment of FAEW or fluoxetine. In addition, the transcriptional levels of *NRF2* and *KEAP1* were not affected by the treatment of FAEW or fluoxetine. This can be taken as another hint for the capability of FAEW and fluoxetine to inhibit the KEAP1-NRF2 protein-protein interaction. Furthermore, we performed molecular docking to investigate the binding modes of the identified FAEW compounds with KEAP1. Indeed, oroxyloside, baicalin, liquiritin, paeoniflorin, albiflorin etc. revealed strong affinities to KEAP1, which might be the effective substances of FAEW against oxidative stress. Importantly, some compounds have been demonstrated to pass the blood-brain barrier and to reach the brain tissue (*e.g*. albiflorin, paeoniflorin and liquiritin), which may further explain the effect of FAEW on the central nervous system^33^.

In this study, we proved that FAEW exerted strong activity against oxidative stress in human glioblastoma T98G and human neuroblastoma SH-SY5Y cell lines. The herbal mixture reduced both endogenous and exogenous ROS, strongly promoted NRF2 translocation to the nucleus in a KEAP1-dependent manner and, hence, increased HO-1 levels. The underlying mechanism for FAEW against oxidative stress may be related to multiple compounds, which bound to KEAP1-NRF2 protein-protein interaction sites and leaded to the release of NRF2 from KEAP1 and NRF2 translocation to the nucleus, implying FAEW might be clinically active against psychiatric diseases through its strong anti-oxidative effect. In a related pervious study in which published gene expression profiles from PTSD patients were used as the starting point, we demonstrated that one or several chemical constituents of FAEW inhibited the NFκB pathway^34^. While the *in vitro* results presented here highlight the role of KEAP1-NRF2 signaling, the interplay of oxidative stress and inflammation as well as the cross-talk between NFκB pathway and NRF2 pathway has been amply demonstrated for several pathological conditions^35, 36^. On the one hand, future studies, should therefore focus on the individual roles of the identified compounds from FAEW and their binding modes to the proteins involved. On the other hand, more *in vivo* studies and clinical trials should be performed to validate the multiple targets of FAEW with respect to psychiatric disorders such as PTSD and to explore the underlying mechanisms.

## Methods

### Chemicals and extracts

FAEW extract was prepared from pills (*Xiaoyao wan*) purchased from Wanxi Pharmaceutical Company (Henan Province, China). Pills were dissolved with the solvents H_2_O: MeOH: DCM = 1:4:5 for three days. A rotary evaporator was used to remove the solvents and the final extract was stored at −20 °C. Fluoxetine was purchased from Sigma-Aldrich (Steinheim, Germany). Both FAEW and fluoxetine were dissolved in distilled water as stock solution for biological experiments. 2′,7′-Dichlorodihydrofluorescein diacetate (H_2_DCFH-DA) was purchased from Sigma-Aldrich (Steinheim, Germany). The chemical composition of the same batch of FAEW used in the present investigation was recently reported^34^.

### Cell cultures

Human glioblastoma T98G cell line was obtained from the German Cancer Research Center (DKFZ, Heidelberg, Germany). Human neuroblastoma SH-SY5Y cell line was obtained from University Medical Center of the Johannes Gutenberg University (Mainz, Germany). The original source of these two cell lines is the American Type Culture Collection (ATCC). Human glioblastoma T98G cell line was cultivated under standard conditions (37 °C, 5% CO_2_) in DMEM medium supplemented with 10% fetal bovine serum (Life Technologies, Darmstadt, Germany), 1% penicillin/streptomycin (Life Technologies, Darmstadt, Germany). Human neuroblastoma cell line SH-SY5Y was cultivated under standard conditions (37 °C, 5% CO_2_) in DMEM/F-12 (without phenol red, with 1% glutamine, Life Technologies, Darmstadt, Germany) supplemented with 10% fetal bovine serum. Cells were passaged twice a week. All experiments were performed with logarithmically growing cells.

### Cell viability assay

Cell viability was evaluated by resazurin assay. One hundred microliters of cell suspension with 5000 or 10000 cells per well (T98G cells: 5000 cells per well; SH-SY5Y cells: 10000 per well) were seeded into 96-well plates one day before the treatment with different concentrations of FAEW and fluoxetine. All these different concentrations of drugs were diluted with 200 fold medium from the stock solution. Distilled water was used as solvent control with the same dilution ratio. After 48 h, 20 µl resazurin (Sigma-Aldrich, Steinheim, Germany) 0.01% w/v in ddH_2_O was added to each well and the plates were incubated at 37 °C for 4 h. The fluorescence was measured with an Infinite M200 Proplate Reader (Tecan, Crailsheim, Germany) using an excitation wavelength of 544 nm and an emission wavelength of 590 nm. The toxicity of compounds was determined by means of the formula:

$$\begin{array}{c}{\rm{Cell}}\,{\rm{Viability}}\,( \% \,{\rm{of}}\,{\rm{control}})=\frac{{\rm{Absorption}}\,{\rm{from}}\,{\rm{sample}}\,{\rm{well}}-{\rm{absorption}}\,{\rm{from}}\,{\rm{medium}}}{{\rm{Absorption}}\,{\rm{from}}\,{\rm{solvent}}\,{\rm{treated}}\,{\rm{cells}}-{\rm{absorption}}\,{\rm{from}}\,{\rm{medium}}}\times 100\end{array}$$

The calculated cell viability (y-axis) was plotted against the log drug concentration (x-axis) using Microsoft Excel.

### Measurement of reactive oxygen species with flow cytometry

H_2_DCFH-DA (Sigma-Aldrich, Steinheim, Germany) is an indicator dye used for the highly sensitive and quantifiable detection of ROS. H_2_DCFH-DA is cleaved by cytoplasmic esterases into 2′,7′-dichlorodihydrofluorescein (H_2_DCF), if it diffuses into the cells. In the presence of hydrogen peroxide, H_2_DCF is oxidized to the fluorescent molecule dichlorofluorescein (DCF) by peroxidases. The fluorescent signal emanating from DCF can be measured and quantified by flow cytometry^37–39^. Briefly, 2 × 10^5^/well T98G cells or 4 × 10^5^/well SH-SY5Y cells were cultured in 6-well plates, and FAEW extract and fluoxetine with different concentrations were added to the medium the day after the cells attached. Distilled water was used as solvent control. After 24 h incubation, the medium was removed and the cells were washed for three times with PBS, 10 μM H_2_DCFH-DA was incubated for 30 min in the dark at 37 °C. Subsequently, the cells were washed with PBS for three times and resuspended in PBS containing H_2_O_2_ (200 μM) (Sigma-Aldrich, Steinheim, Germany) or only PBS, respectively, for 15 min. The samples were immediately measured in a FACS Calibur flow cytometer (Becton-Dickinson, Heidelberg, Germany). For each sample, 1 × 10^4^ cells were counted. DCF was measured at 488 nm excitation and detected using a 530/30 nm bandpass filter. All parameters were plotted on a logarithmic scale. Cryptographs were analyzed using FlowJo software (Celeza). All experiments were performed at least in triplicate.

### Microarray gene expression profiling

T98G cells were treated with 200 µg/ml FAEW and 20 µM fluoxetine or distilled water as solvent control for 48 h, and H_2_O_2_ (200 µM) was added to induce oxidative stress for 6 h before total RNA was isolated using InviTrap spin Universal RNA Mini kit (250) (STRATEC Molecular, Berlin, Germany) according to the manufacturer’s instruction. RNA concentrations were determined using a nanodrop spectrophotometer (Nanodrop Technologies, Thermo Fisher, Dreieich, Germany). Microarray hybridizations were performed in duplicate for treated samples and for control samples by the Genomics and Proteomics Core Facility at the German Cancer Research Center (DKFZ, Heidelberg, Germany). A detailed protocol has been previously published by us^40^. Data analysis was done by using the quantile normalization algorithm without background subtraction, and differentially regulated genes were defined by calculating the standard deviation differences of a given probe in a one-by-one comparison of samples or groups. The data obtained was further filtered with Chipster software including the steps filtering of genes by two times standard deviation and a subsequent assessment of significance using empirical Bayes t-test (*p* < 0.05) with Bonferroni correction. Filtered genes were analyzed by the Ingenuity Pathway Analysis software (IPA, Ingenuity Systems, Redwood, CA, USA) to determine cellular networks and functions affected by each drug treatment. These results were further processed by using the comparison analysis tool, offering the possibility to compare datasets of samples treated by different compounds. Each gene which appeared upon treatment of FAEW or fluoxetine was compared with the H_2_O_2_-induced stress control or non-induced solvent control, respectively. The H_2_O_2_ group was compared with a non-induced solvent control.

### Real-time reverse transcription-PCR

Real-time RT-PCR was performed with the same samples used for microarray experiments. Total RNA samples were converted to cDNA with random hexamer primers by RevertAid H Minus First Strand cDNA Synthesis Kit (Thermo Scientific, Darmstadt, Germany). Oligonucleotides were synthesized by Eurofins MWG Operon (Ebersberg, Germany). The efficiency of all primer pairs used for real-time PCR expression was better than 90%. Quantification of cDNA was performed on CFX384 Real-Time PCR Detection System (Bio-Rad, München, Germany) using a Hot Start Taq EvaGreen qPCR Mix (Axon scientific, Göttingen, Germany). RT-PCR was performed with an initial denaturation at 95 °C for 10 min followed by 40 cycles including strand separation at 95 °C for 15 s, annealing at 57.5 °C for 40 s and extension at 72 °C for 1 min. After PCR product amplification, melting curves were computed. The expression levels were normalized to the transcription level of the housekeeping gene, *RPS13*. All samples were run in duplicates and the experiment was repeated once.

### Western blotting

T98G cells were treated with FAEW (200 µg/ml) or fluoxetine (20 µM) for 48 h. Before protein extraction, 200 µM H_2_O_2_ was added for 24, 12, 6 h or 10 min. Nuclear and cytoplasmic protein extracts were prepared according to the NE-PER nuclear and cytoplasmic extraction reagent (Thermo Scientific) supplemented with EDTA-free Halt Protease Inhibitor Cocktail (Thermo Scientific). Total protein extracts were extracted with M-PER™ Mammalian Protein Extraction Reagent (Thermo Scientific, Darmstadt, Germany). Besides, Nuclear and cytoplasmic protein extracts were prepared after T98G and SH-SY5Y cells were treated with different concentrations of FAEW (600 µg/ml, 200 µg/ml, 60 µg/ml, 20 µg/ml) and fluoxetine (20 µM, 6 µM, 2 µM). Protein concentrations were measured with Nanodrop 1000 spectrophotometry (Thermo Scientific, Darmstadt, Germany). The densities of the protein bands were quantified by FluorChemQ software (Biozym Scientific Company, Oldendorf, Germany). Nuclear NRF2 levels were determined with anti-NRF2 monoclonal antibody (1:3000, Cell signaling, Frankfurt, Germany). TATA-binding protein (TBP) and Lamin A/C levels served as the internal control for nuclear protein, using the anti-TBP monoclonal antibody or anti-Lamin A/C polyclonal antibody (1:3000, Cell Signaling, Frankfurt, Germany). β-actin served as the internal control for total and cytoplasmic protein using the anti-β-actin monoclonal antibody (1:3000, Cell Signaling, Frankfurt, Germany).

### Isolation of compounds from FAEW and structure identification

The FAEW extract (2 g) was dissolved in DMSO and bound to 2 g of C18 material (Merck Lichroprep RP-18, 25–40 µm), which was then dried by lyophilization. Solid phase extraction (Agilent MegaBE-C18, 10 g) was performed by using a step gradient. The first fraction was eluted with 100% H_2_O and discarded. Intermediate I (227.5 mg) was eluted with 15% MeCN and intermediate II (81.9 mg) with 30% MeCN. Preparative HPLC with intermediate I (MeCN/H_2_O with 0.1% TFA gradient, 5% MeCN to 25% MeCN in 40 min, 21.2 ml/min, Agilent Eclipse XDB-Phenyl, 5 µm, 21.2 × 150 mm) yielded FAEW-1 (3.2 mg, RT 18–18.5 min), FAEW-2 (18.3 mg, RT 18.75–19.5 min), FAEW-3 (6.4 mg, RT 27.75 min), and FAEW-4 (1.1 mg, RT 36 min). FAEW-5 (2.3 mg, RT 15.5 min), FAEW-6 (1.9 mg, RT 20.6 min), FAEW-7 (2.1 mg, RT 22.3 mg), FAEW-8 (1.3 mg, RT 27.3 min), and FAEW-9 (1.0 mg, RT 31.8 min) were isolated by semi-preparative HPLC with intermediate II (MeCN/H_2_O with 0.1% TFA gradient, 17% MeCN to 33.2% MeCN in 38 min, 4 ml/min, Agilent Eclipse XDB-Phenyl, 5 µm, 9.4 × 250 mm). NMR and MS methods were used to identify the structure of the isolated compounds, the method and results were attached as supplementary information.

### Molecular docking

Ten compounds identified in the FAEW extract (albiflorin, paeoniflorin, baicalin, 1- (2, 4-dihydroxyphenyl)-3-hydroxyl-3-(4-hydroxyphenyl)-1-propanone (β-hydroxy-DHP), ononin, isoliquiritin, isoliquiritin apioside, liquiritin, oroxyloside, pentagalloyl-β-D-glucose) were selected for the *in silico* molecular docking analyses together with the anti-depressant drug fluoxetine and the known KEAP1 inhibitor, *N*,*N*’-naphthalene-1,4-diylbis (4-methoxybenzenesulfonamide)^41^ (PubChem ID:IQK) in order to compare their binding affinity and docking poses on KEAP1. The PDB structure file of the protein was downloaded from RCSB Protein Data Bank (http://www.rcsb.org/pdb/home/home.do). All bound water molecules and ligands were eliminated from the protein and polar hydrogen was added. 3D structures of these compounds were downloaded from PubChem, ChemSpider was used to convert mol files to pdb file after checking absolute and relative configuration. Molecular docking was then carried out with AutoDock 4.2 (The Scripps Research Institute, La Jolla, CA) following a protocol previously reported by us^42^. Docking parameters were set to 250 runs and 25,000,000 energy evaluations for each cycle. VMD (Visual Molecular Dynamics) was used for visualization of the binding modes obtained from docking. The average of the lowest binding energy of three runs was taken into account.

### Literature search

The PubMed database was searched with the corresponding compound name and ROS as key words. Identified literature was classified into *in vitro*, *in vivo* to give a retrospective summary of the current state of their activity towards oxidative stress.

### Statistical analysis

Unless otherwise indicated, data are expressed as means ± SD of three independent experiments. Student’s t tests were used to compare the means of two groups. *P*-value < 0.05 was considered as significant.

## Electronic supplementary material

Supplementary information
