# Supplementary material for: The Chinese herbal formula Free and Easy Wanderer ameliorates oxidative stress through KEAP1-NRF2/HO-1 pathway
Source: Sci Rep. 2017 Sep 14;7:11551. doi: 10.1038/s41598-017-10443-6 (PMC5599498; doi:10.1038/s41598-017-10443-6)
Supplement: Supplementary file 1 — Supplementary information [file 41598_2017_10443_MOESM1_ESM.pdf]

The Chinese herbal formula *Free and Easy Wanderer* ameliorates oxidative stress through the KEAP1-NRF2/HO-1 pathway

Authors: Chunlan Hong[1,6], Jingming Cao[1], Ching-Fen Wu[1], Onat Kadioglu[1], Anja Schüffler[2], Ulrich Kauhl[3], Sabine M. Klauck[4], Till Opatz[3], Eckhard Thines[2], Norbert W. Paul[5,6], Thomas Efferth[1*]

Affiliations:

1 Department of Pharmaceutical Biology, Institute of Pharmacy and Biochemistry, Johannes Gutenberg University, Staudinger Weg 5, 55128 Mainz, Germany

2 Institute of Biotechnology and Drug Research, Kaiserslautern, Germany, and Institute of Biotechnology, Johannes Gutenberg University, Mainz, Germany.

3 Institute of Organic Chemistry, Johannes Gutenberg University, Mainz, Germany.

4 Division of Cancer Genome Research, German Cancer Research Center (DKFZ), German Cancer Consortium (DKTK), and National Center for Tumor Diseases (NCT), Heidelberg, Germany.

5 Institute of History, Theory and Ethics of Medicine, Johannes Gutenberg University Medical Center, Mainz, Germany

6 DFG Research Training Group GRK 2015/1, Johannes Gutenberg University Medical Center, Mainz, Germany

*** Corresponding author:**

Tel: 49-6131-3925751;

Fax: 49-6131-3923752;

Email: efferth@uni-mainz.de

Running Title: Chinese FAEW formula ameliorates oxidative stress

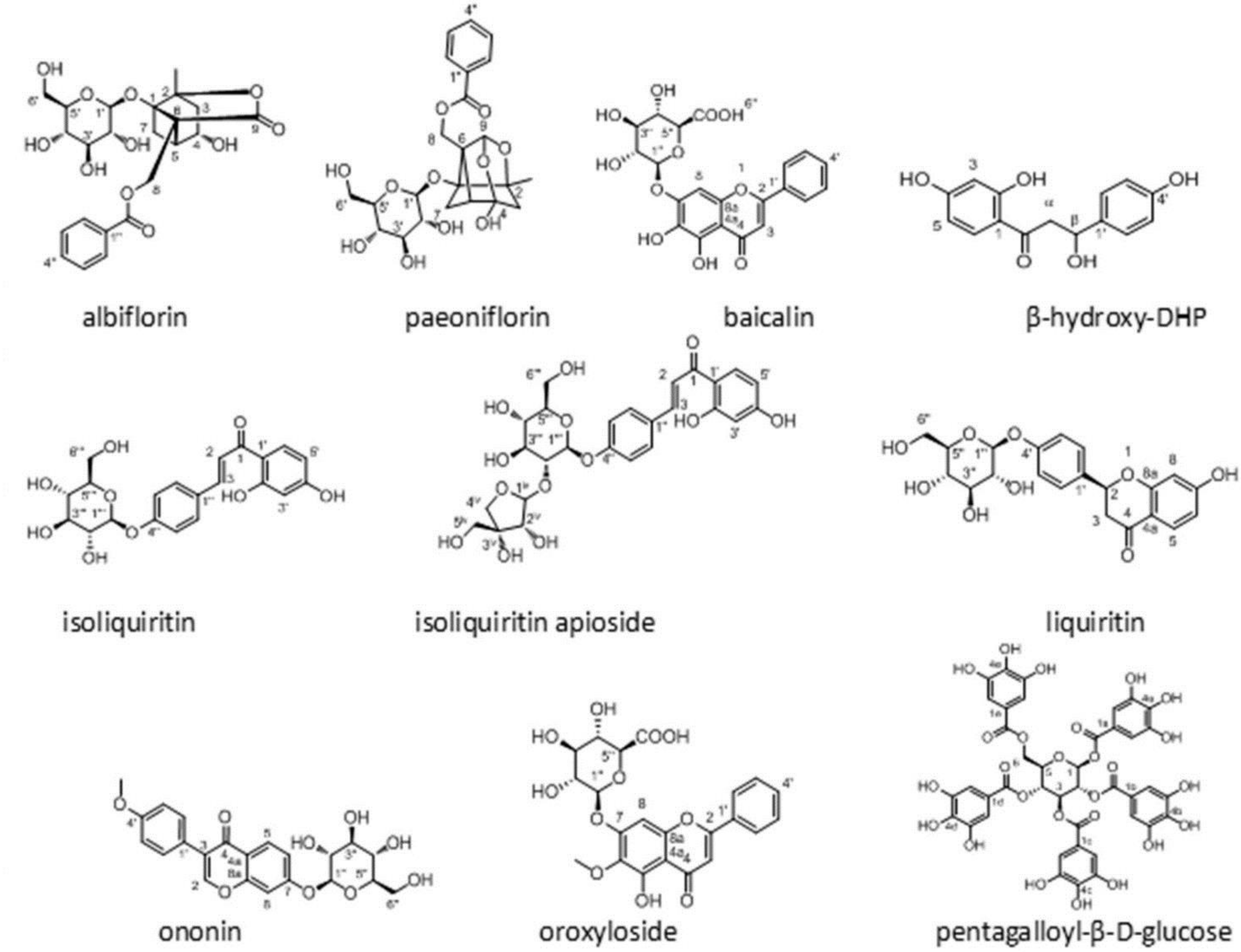

Supplementary Fig. S1. The structures of 10 compounds isolated from FAEW.

Supplementary Table S1. *In silico* molecular docking of chemical compounds isolated from FAEW to Kelch-like ECH-associated protein 1 (KEAP1).

Ligands	Binding energy (kcal/mol)	Ki (μM)	Residues forming H-bonds	Residues involved in hydrophobic interactions
Baicalin	-7.45±0.04	3.47±0.23	Asn382, Asn414, Arg415, Ser555, Ser602	Tyr334, Arg380, Ile461, Gly462, Phe478, Ser508, Ala556, Tyr572
Ononin	-5.57±0.08	83.58±10.40	Ser602	Tyr334, Ser363, Gly364, Arg415, Ile416, Gly462, Ala556, Tyr572, Phe577, Gly603
Isoliquiritin	-5.87±0.19	51.78±15.65	Asn382, Ser602	Tyr334, Gly364, Arg415, Ile416, Gly462, Gly509, Ala510, Ala556, Phe577, Gly603
Liquiritin	-6.86±0.06	9.46±0.93	Arg380, Arg415, Ser555	Tyr334, Ser363, Gly364, Asn382, Asn414, Gly462, Arg483, Gly509, Tyr525, Gln530, Ala556, Ser602
Isoliquiritin apioside	-4.42±0.19	592.15±191.29	Tyr334, Asn414, Arg415	Ser363, Asn382, Ile461, Gly462, Arg483, Ser508, Gly509, Tyr525, Tyr572, Phe577, Ser602
β-hydroxy-DHP	-5.32±0.34	139.60±25.36	Asn382	Tyr334, Ser363, Gly364, Arg380, Asn414, Arg415, Ile416, Gly462, Ala556, Gly603
Pentagalloyl-β-D-glucose	-2.68±0.14	11.15±2.51	Asn382, Arg483	Tyr334, Ser363, Arg415, Gly509, Tyr525, Gln528, Asp529, Gln530, Ser555, Ala556, Tyr572, Phe577, Ser602
Oroxyloside	-7.89±0.27	1.73±0.45	Ser363, Arg380, Asn382, Arg415, Ser602	Tyr334, Gly364, Leu365, Asn414, Ile416, Gly462, Gly509, Ala556, Gly603
Albiflorin	-5.93±0.14	45.95±11.73	Asn382	Tyr334, Ser363, Gly364, Arg380, Asn414, Arg415, Arg483, Tyr525,Gln530, Ser555, Ala556, Tyr572, Phe577, Ser602
Paeoniflorin	-6.00±0.08	68.01±5.23	Arg415	Tyr334, Ser363, Asn414, Gly462, Ser508, Gly509, Tyr525, Ala556, Ser602
Fluoxetine (R)	-4.99±0.07	221.02±24.87	Asn382, Asn414, Arg415, Ser555, Ser602	Gly364, Arg415, Gly462, Val463, Ser508, Gly509, Ala510, Tyr525, Ala556, Gly603, Val604
Fluoxetine (S)	-5.01±0.20	220.93±80.57	Ser602	Gly364, Arg415, Ile461, Gly462, Phe478, Arg483, Ser508, Gly509, Ser555, Ala556,
IQK	-9.52±0.18	0.11±0.03	Arg415, Gln530, Ser555	Ser363, Gly364, Asn414, Ile461, Arg483, Ser508, Gly509, Tyr525, Gln530, Ser555, Ala556, Phe577, Ser602, Gly603

Supplementary data

1 Isolation

Standardized HPLC/MS method:

Column:	Agilent XDB Phenyl, 3.5 µm, 150 x 3 mm
Temperature:	40 °C (column oven)
Solvents:	H_2O with 0.1% HCOOH and MeCN
Gradient:	1% MeCN to 50 % MeCN in 40 min
	50% MeCN to 100 % MeCN in 5min
	100% MeCN isocratic for 2 min
	100% MeCN to 1 % MeCN in 1 min
Flow:	0.45 ml/min
MS:	Ion source: APCI
	Capillary voltage: 3500 V (pos.)/2200 V (neg.)
	Corona current: 4.0 µA (pos.)/12 µA (neg.)
	Drying Gas Flow: 6.0 l/min
	Nebulizer pressure: 50 psig
	Drying Gas Temperature: 350 °C
	Vaporizer Temperature: 400 °C

Crude extract "free and easy wanderer" (HPLC-MS chromatogram, 250 nm)

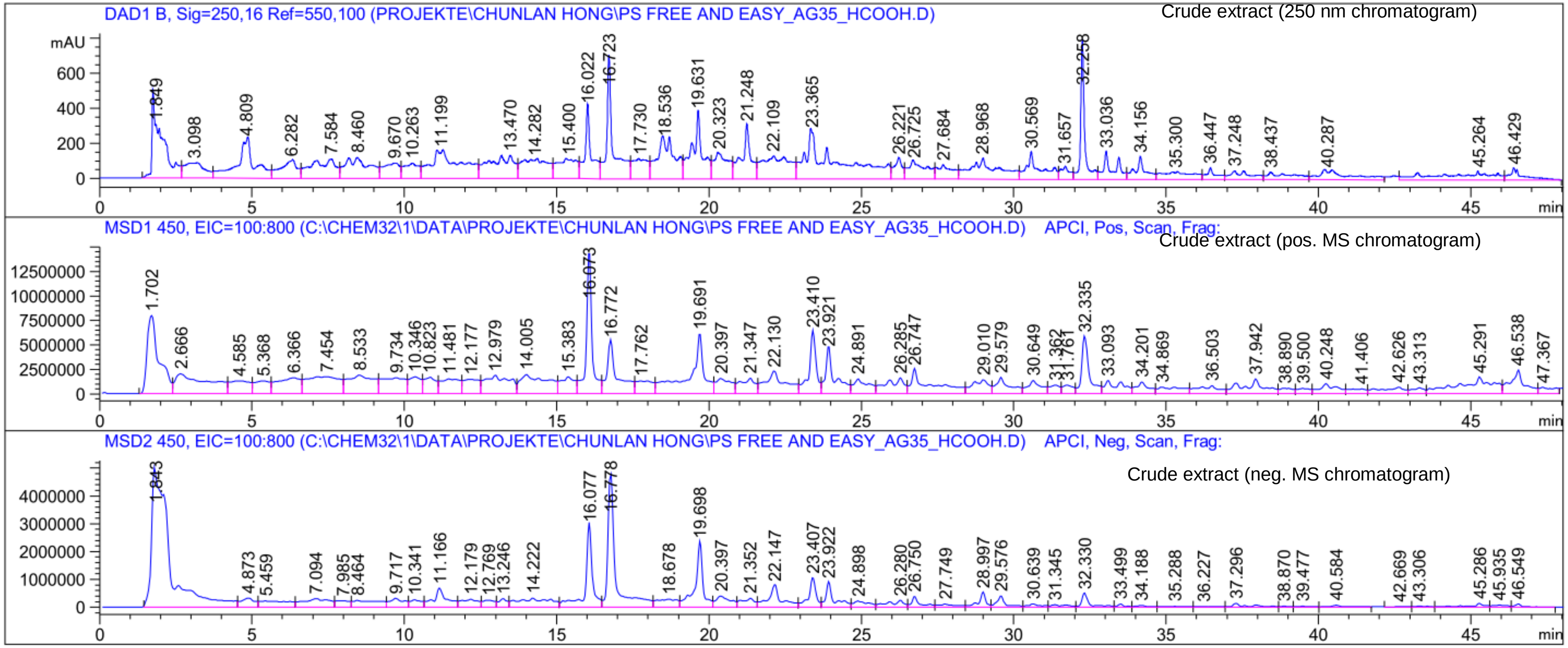

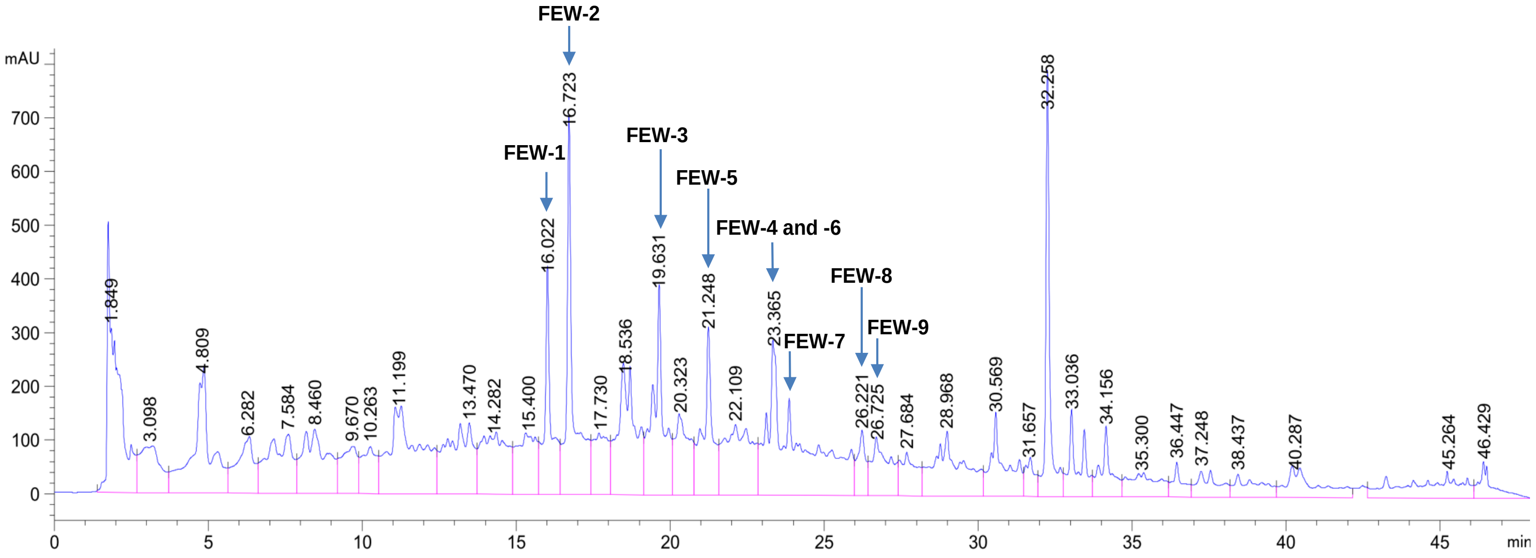

HPLC chromatogram (250 nm) of the crude extract (free and easy wanderer), isolated compounds are marked

1.1. FAEW-1 Albiflorin

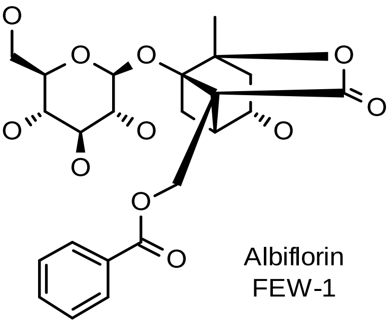

Albiflorin
FEW-1

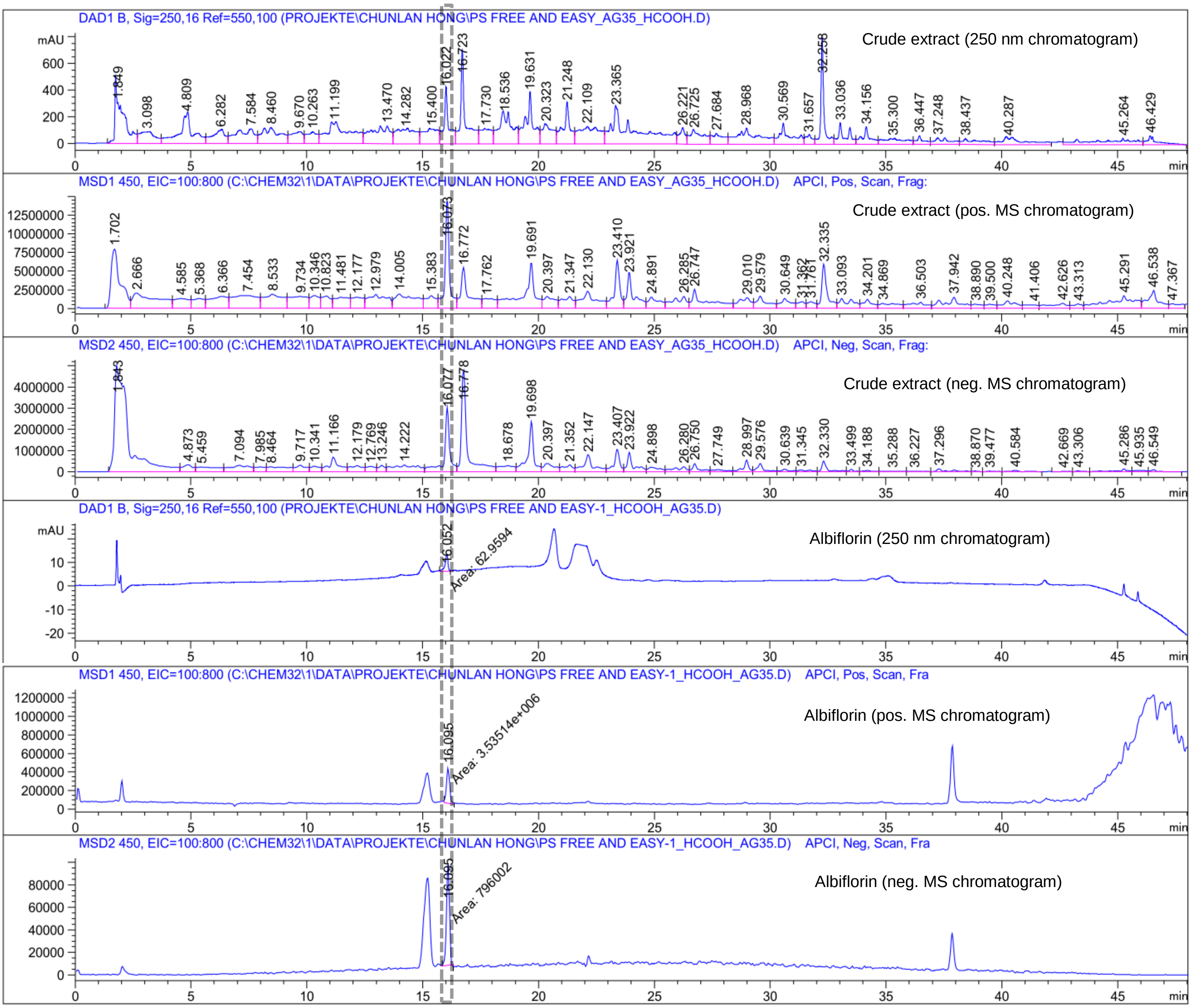

Albiflorin MS-spectra (comparison of corresponding MS spectra)

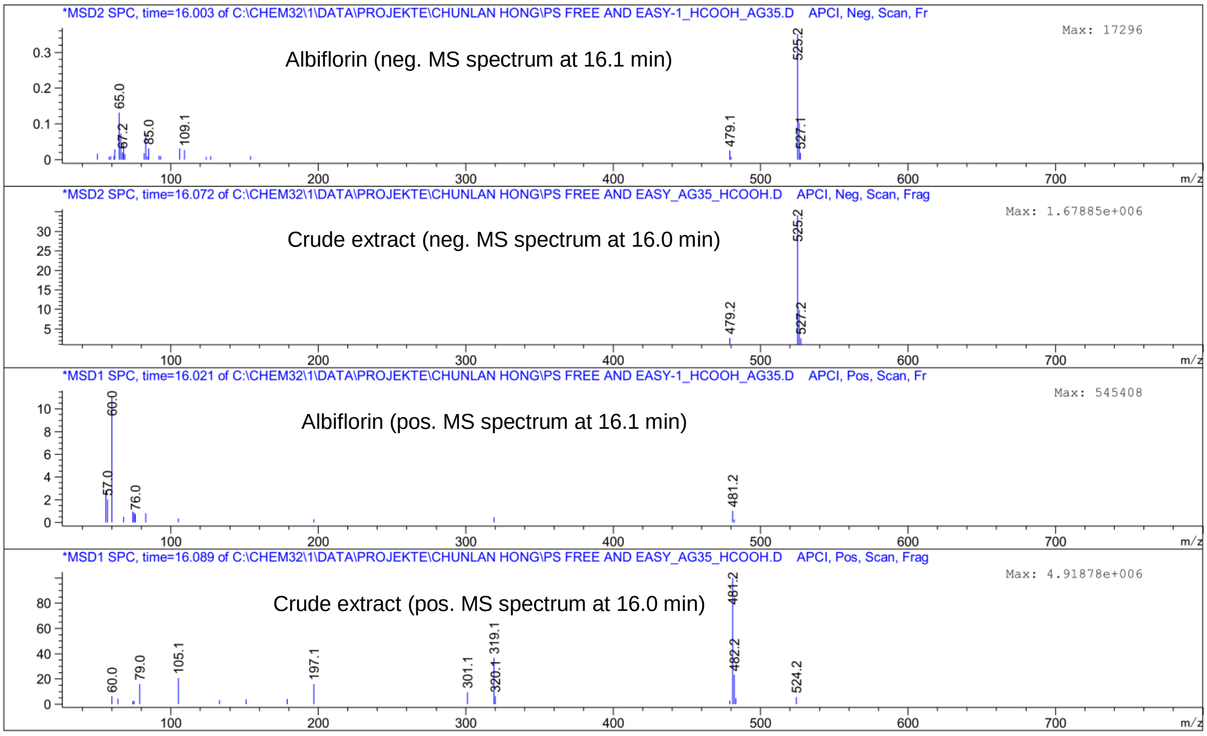

1.2. FAEW-2 Paeoniflorin

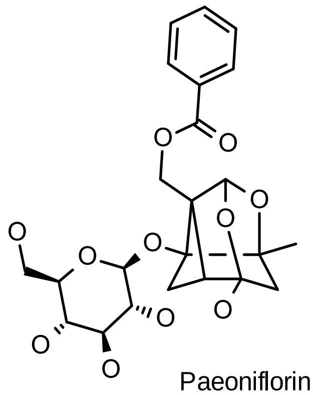

Paeoniflorin
FEW-2

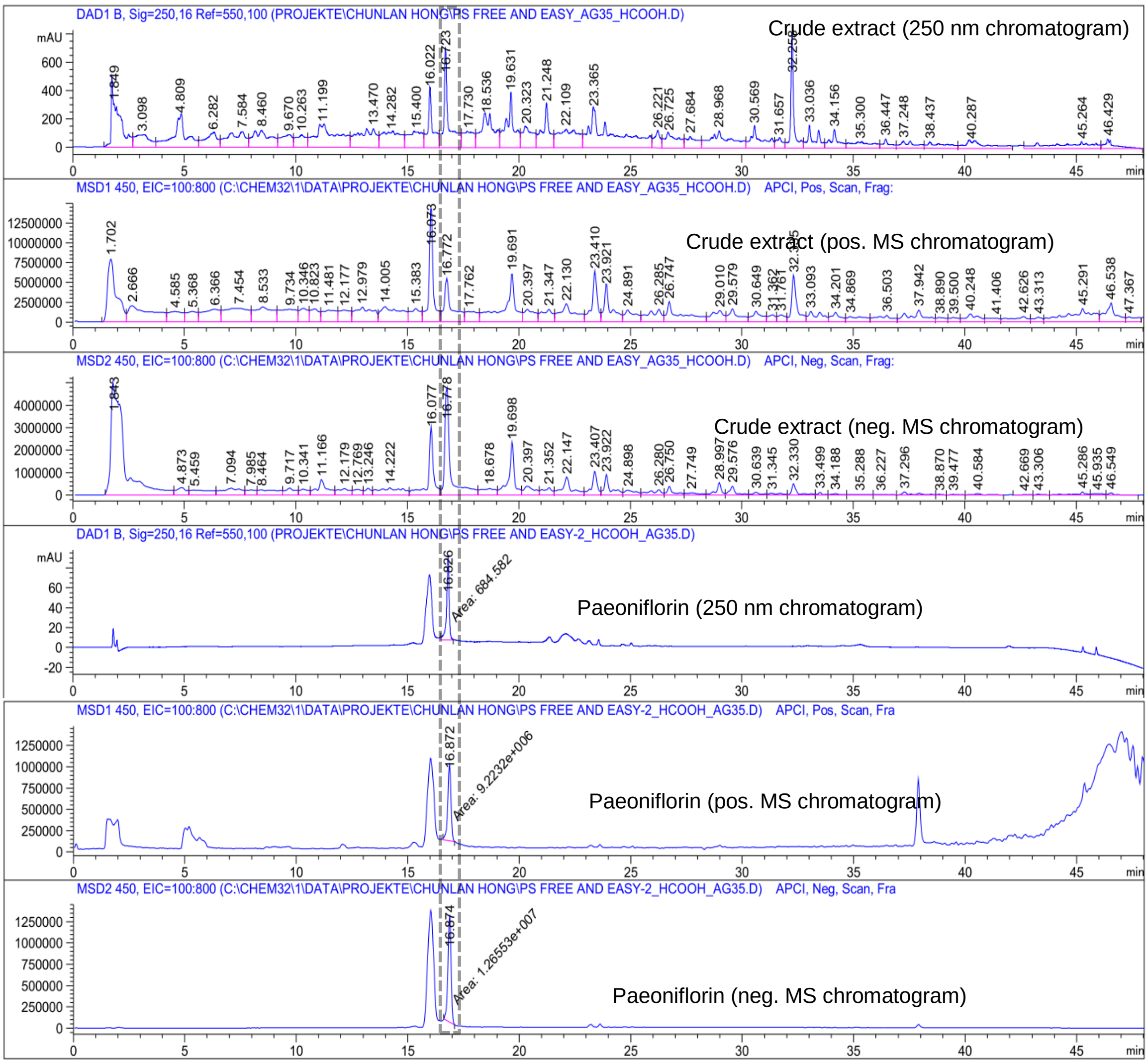

Paeoniflorin: negative MS-spectrum (comparison of corresponding MS spectrum)

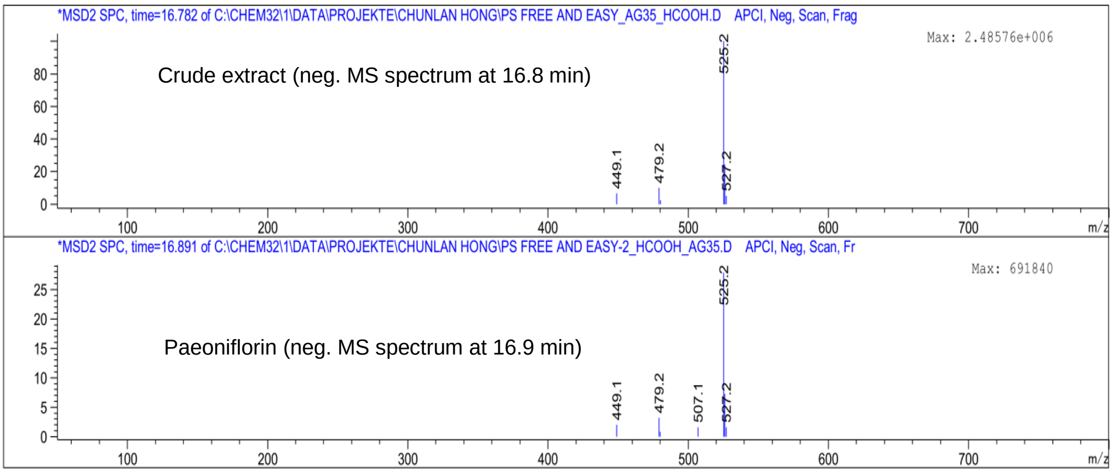

1.3. FAEW-3 Liquiritin

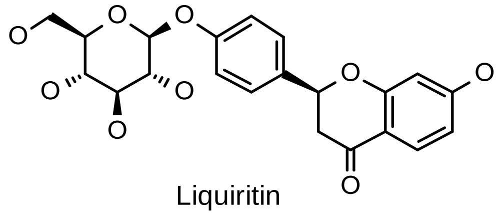

Liquiritin
FEW-3

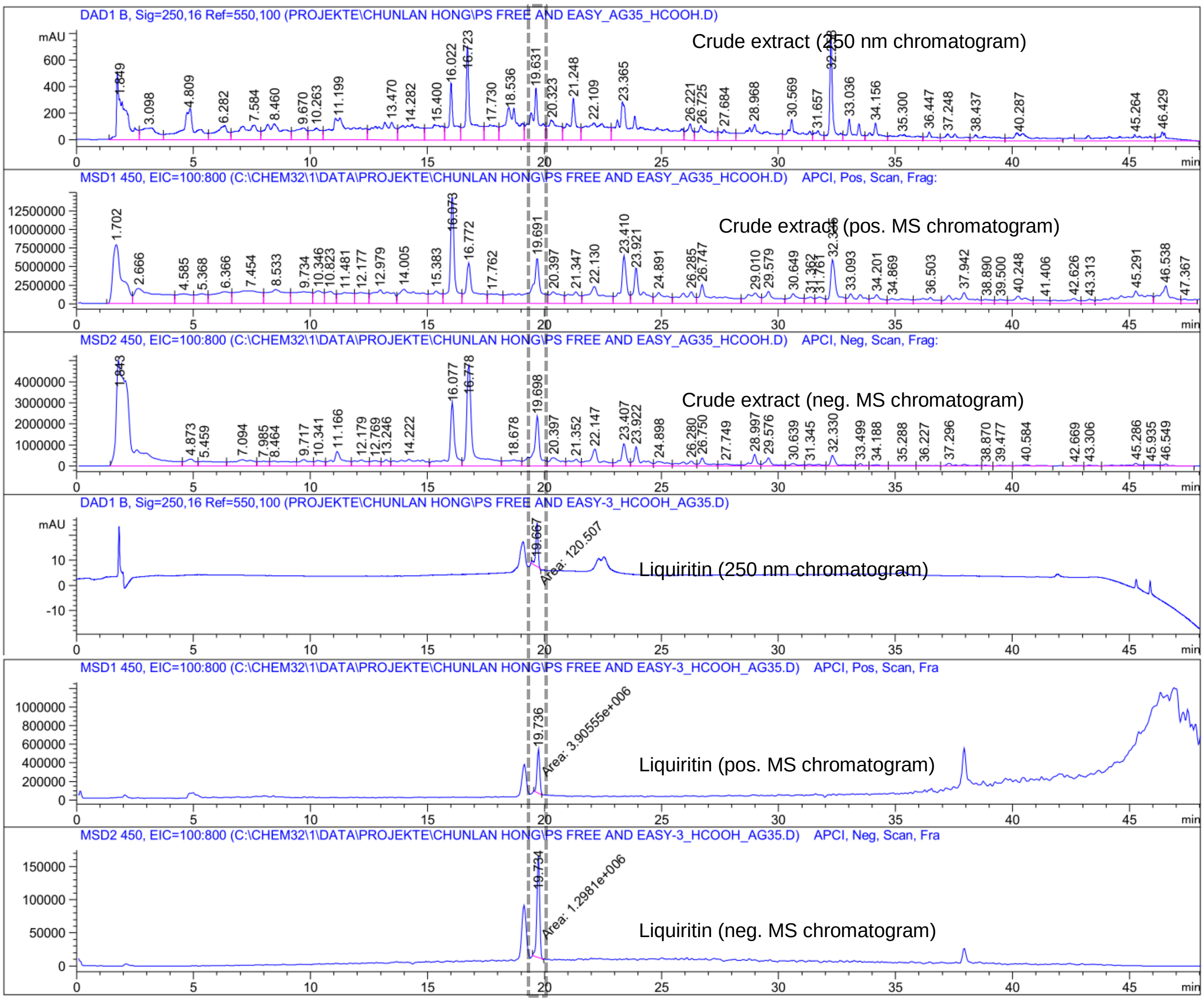

Liquiritin: MS-spectra (comparison of corresponding MS spectra)

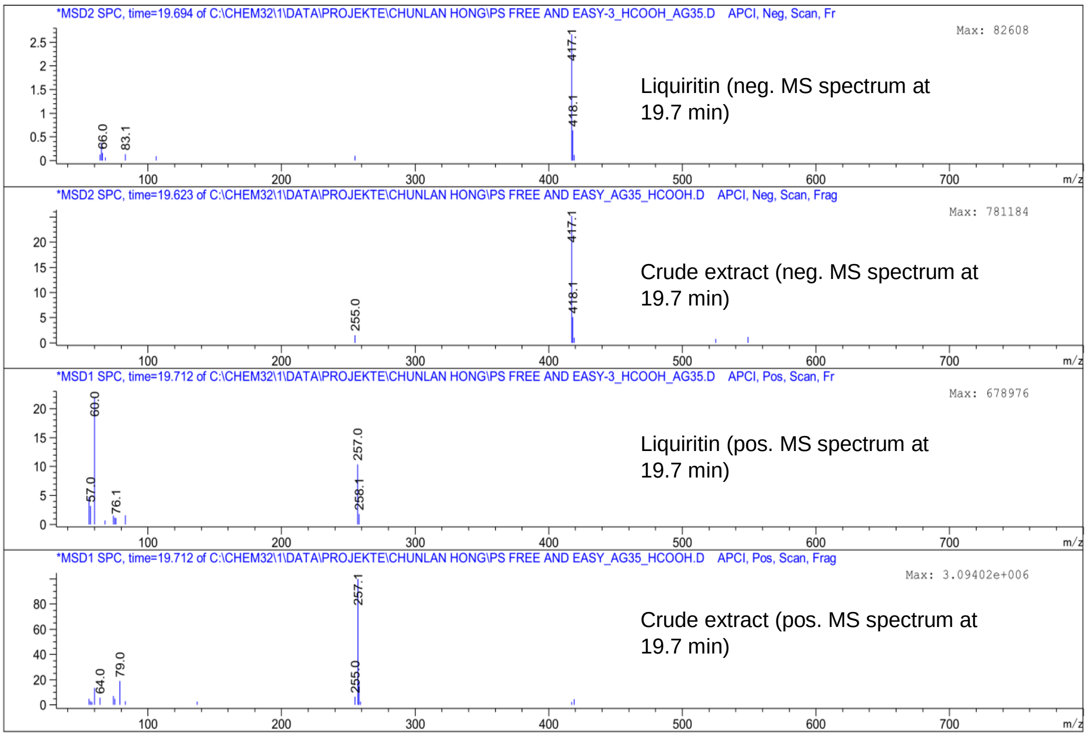
*MSD2 SPC, time=19.694 of C:\CHEM32\1\DATA\PROJEKTE\CHUNLAN HONG\PS FREE AND EASY-3_HCOOH_AG35.D APCI, Neg, Scan, Fr
Max: 82608
Liquiritin (neg. MS spectrum at 19.7 min)
417.1
418.1
66.0
83.1
*MSD2 SPC, time=19.623 of C:\CHEM32\1\DATA\PROJEKTE\CHUNLAN HONG\PS FREE AND EASY_AG35_HCOOH.D APCI, Neg, Scan, Frag
Max: 781184
Crude extract (neg. MS spectrum at 19.7 min)
417.1
418.1
255.0
*MSD1 SPC, time=19.712 of C:\CHEM32\1\DATA\PROJEKTE\CHUNLAN HONG\PS FREE AND EASY-3_HCOOH_AG35.D APCI, Pos, Scan, Fr
Max: 678976
Liquiritin (pos. MS spectrum at 19.7 min)
60.0
57.0
76.1
257.0
258.1
*MSD1 SPC, time=19.712 of C:\CHEM32\1\DATA\PROJEKTE\CHUNLAN HONG\PS FREE AND EASY_AG35_HCOOH.D APCI, Pos, Scan, Frag
Max: 3.09402e+006
Crude extract (pos. MS spectrum at 19.7 min)
257.1
255.0
64.0
79.0
m/z

1.4. FAEW-4 Baicalin

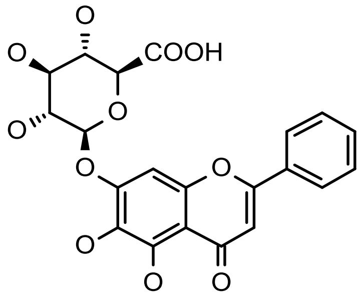

Baicalin
FEW-4

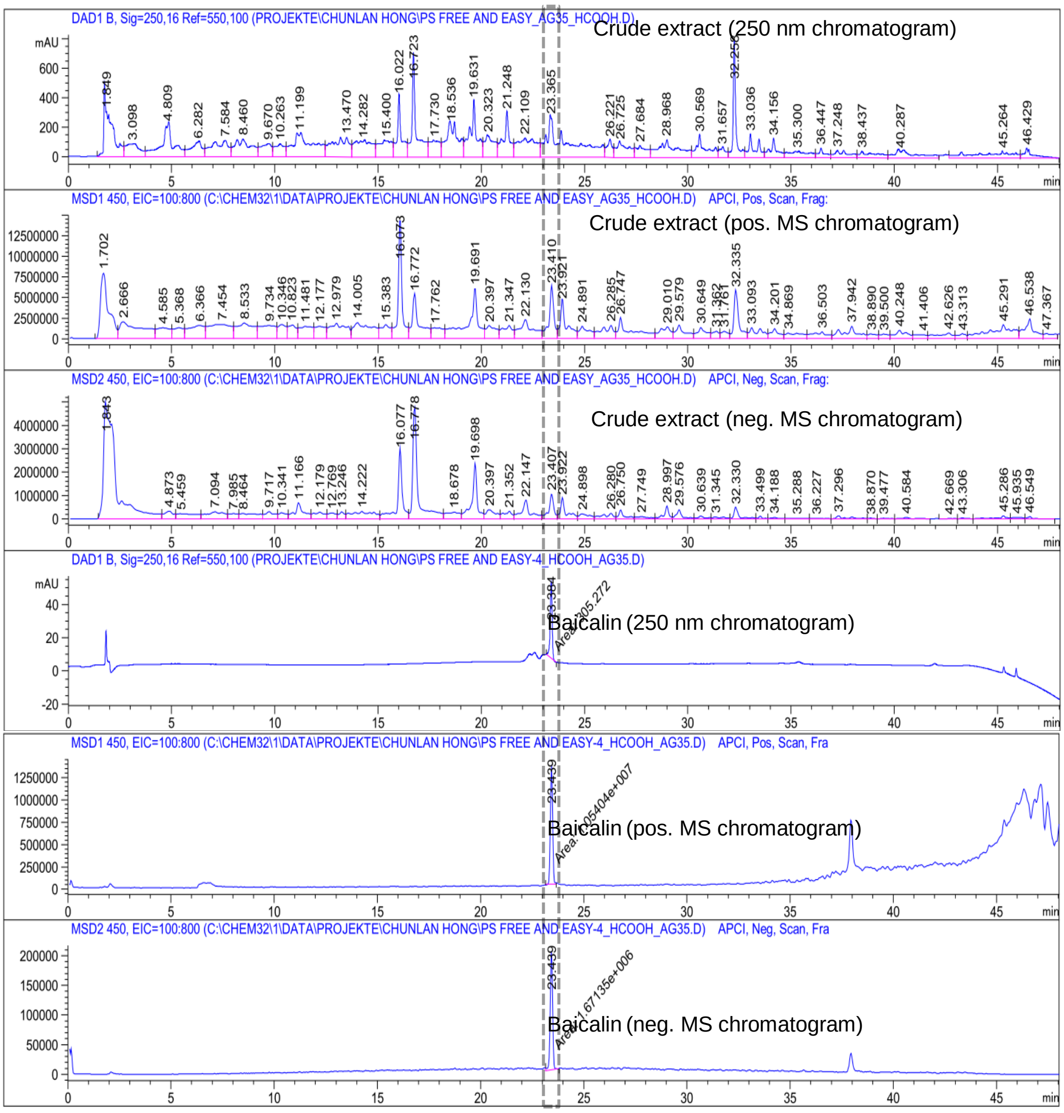

Baicalin: MS-spectra (comparison of corresponding MS spectra)

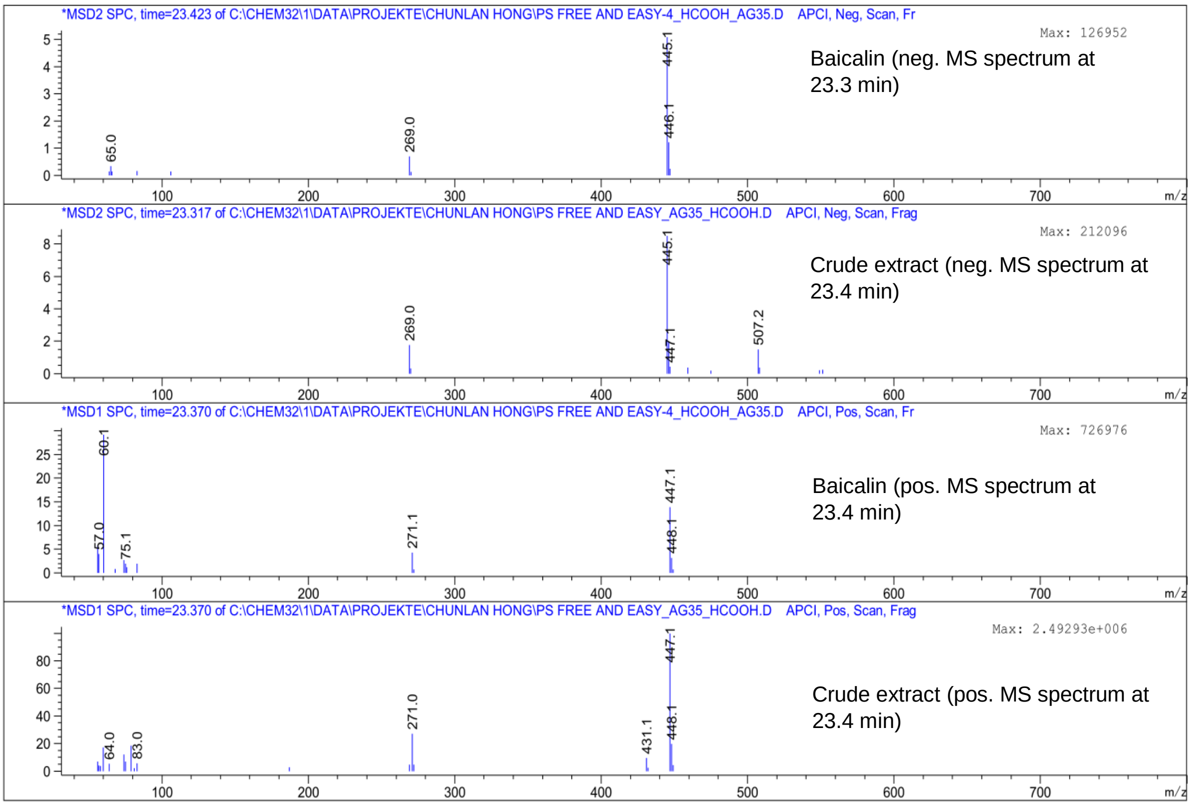

1.5. FAEW-5 Pentagalloyl-glucopyranose

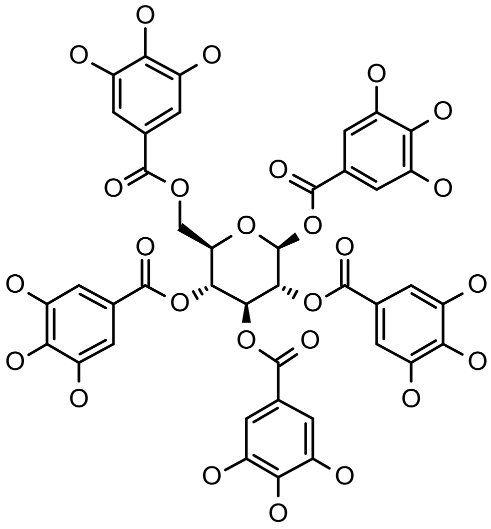

Pentagalloyl-glucopyranose
FEW-5

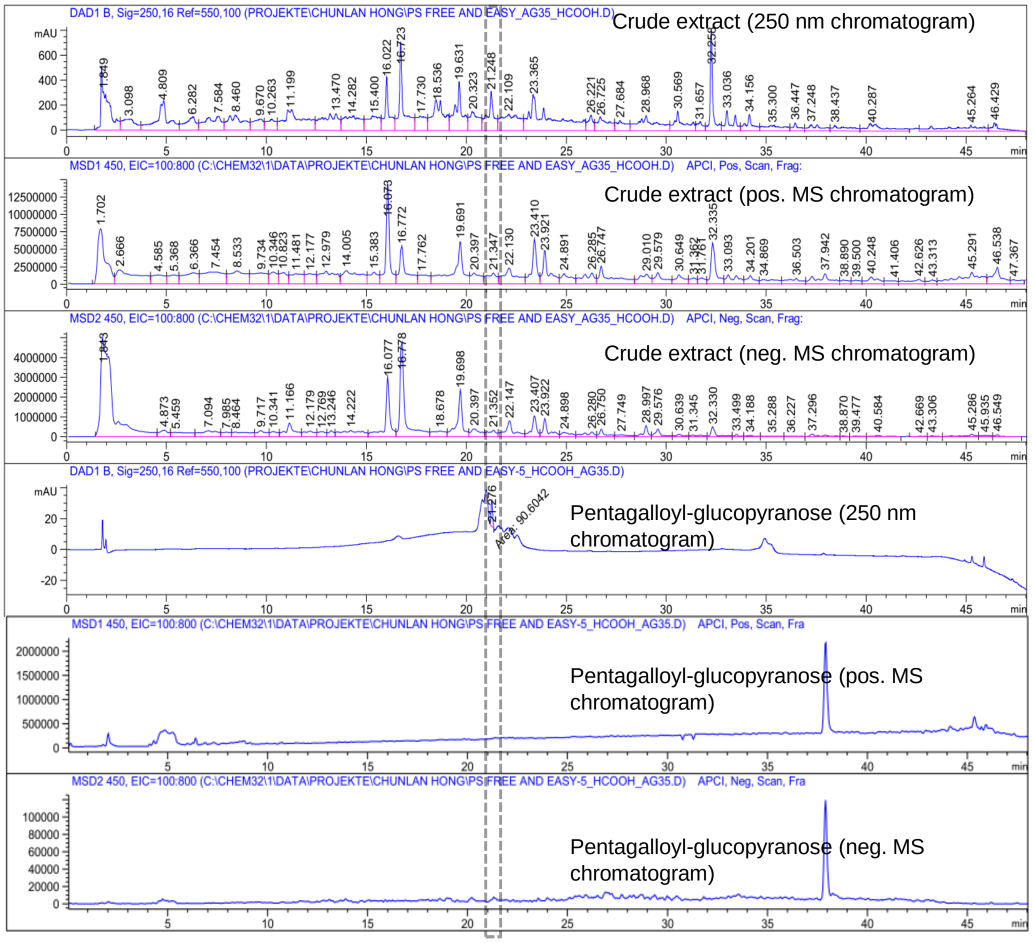

Pentagalloyl-glucopyranose: UV/vis-spectra (comparison of corresponding UV/vis spectra, no mass data was available)

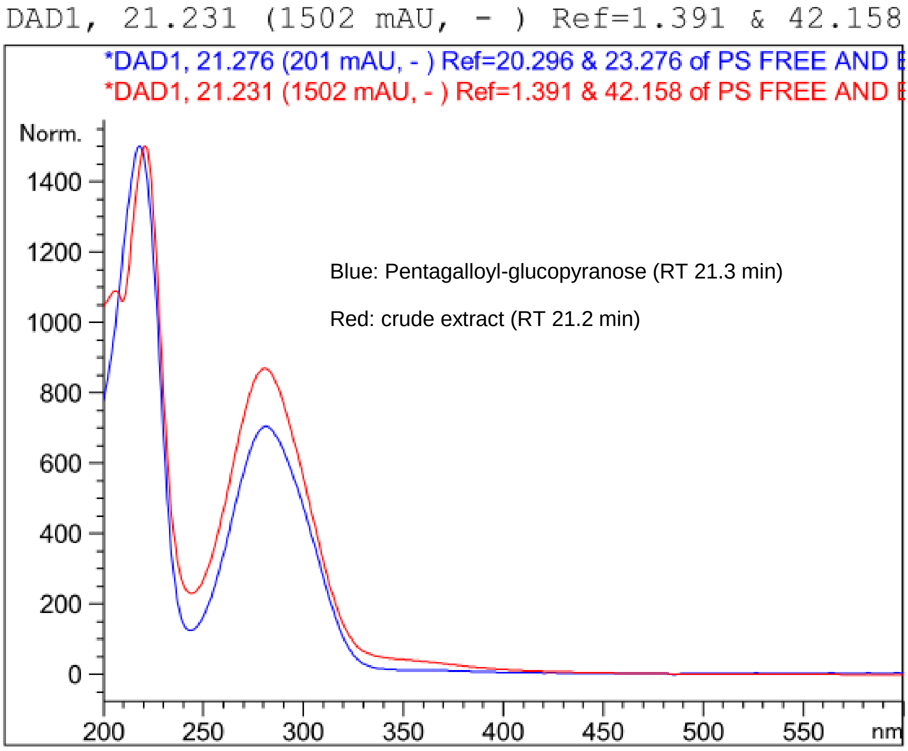

1.6. FAEW-6 Isoliquiritin apioside/Ononin

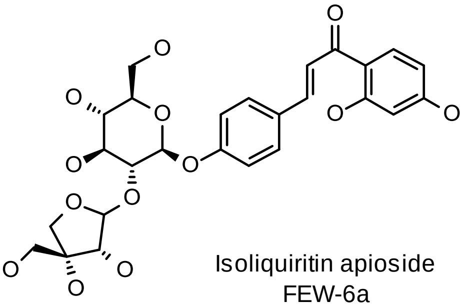

Isoliquiritin apioside
FEW-6a

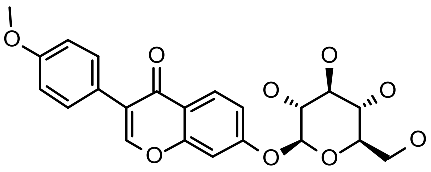

Ononin
FEW-6b

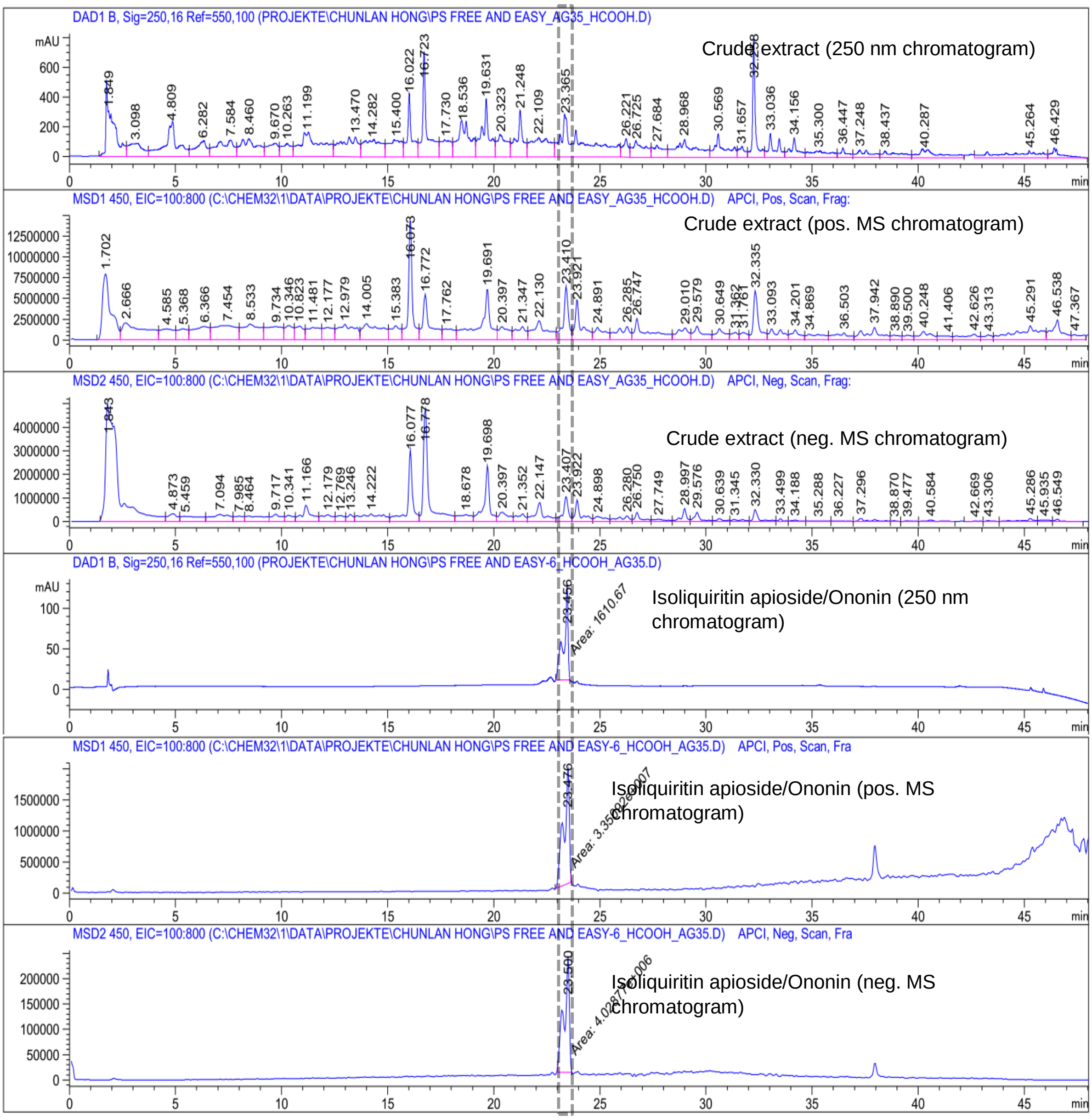

Isoliquiritin apioside/Ononin: negative MS-spectrum (comparison of corresponding MS spectrum)

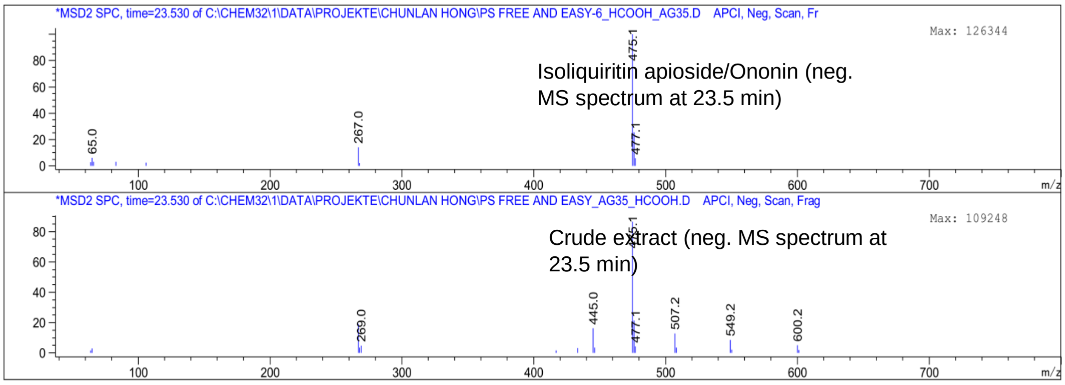

1.7. FAEW-7 Isoliquiritin

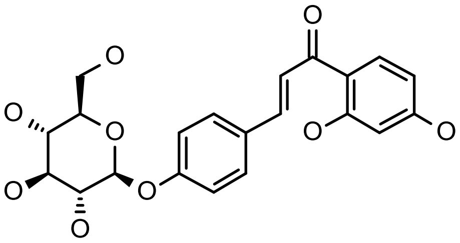

Isoliquiritin
FEW-7

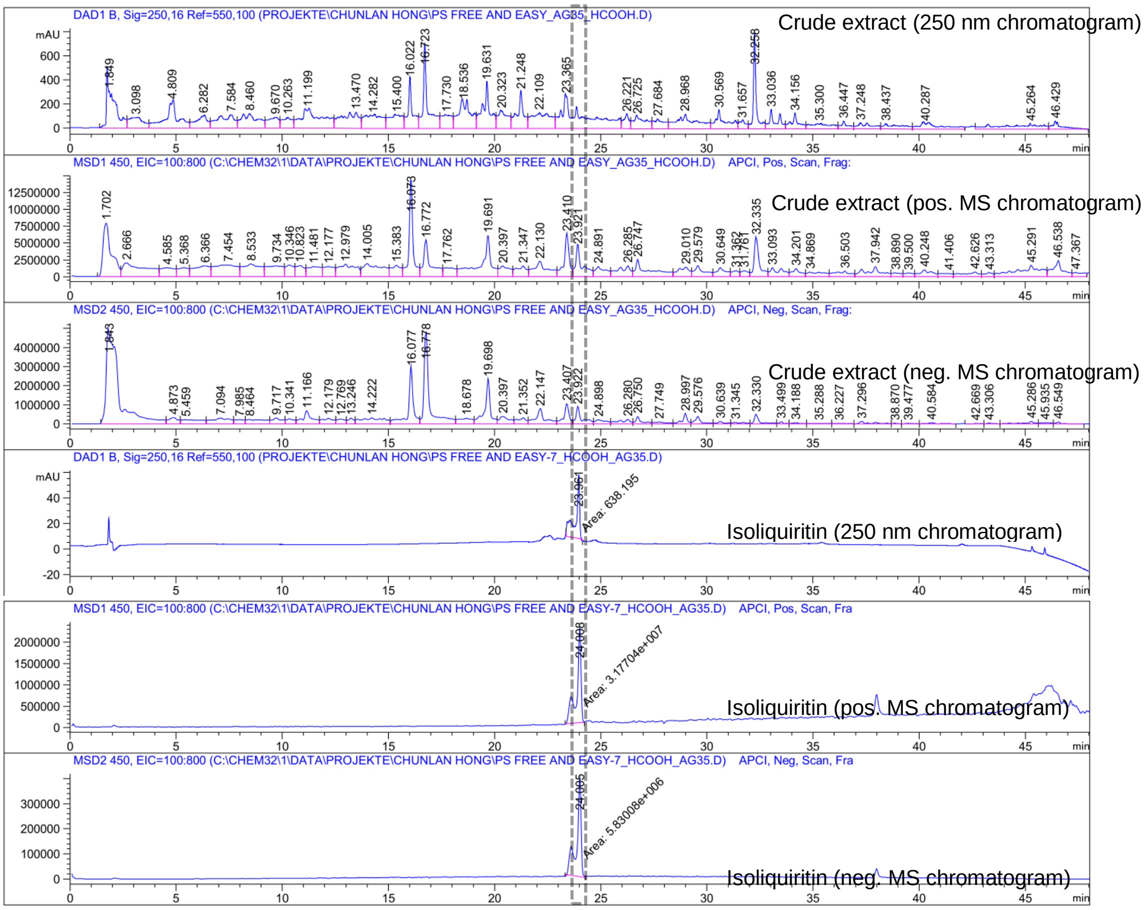

Isoliquiritin: MS-spectra (comparison of corresponding MS spectra)

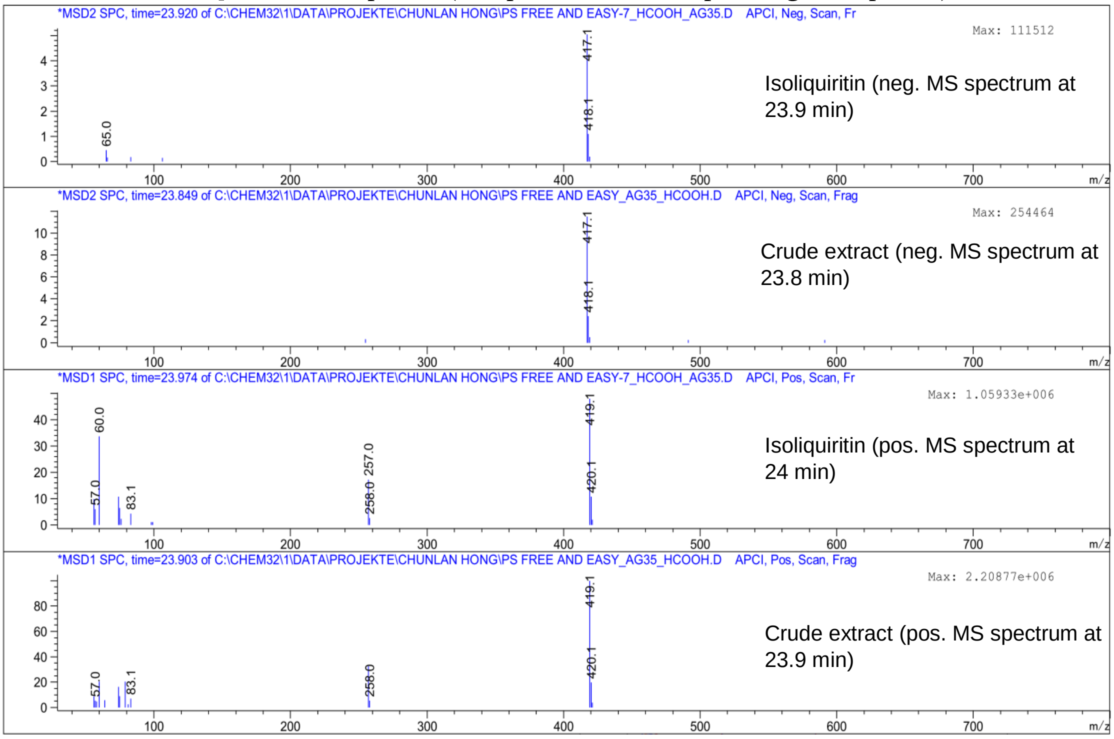

1.8. FAEW-8 ß-Hydroxy-DHP

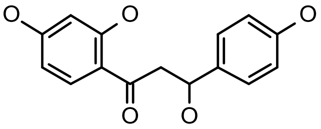

β-Hydroxy-DHP
FEW-8

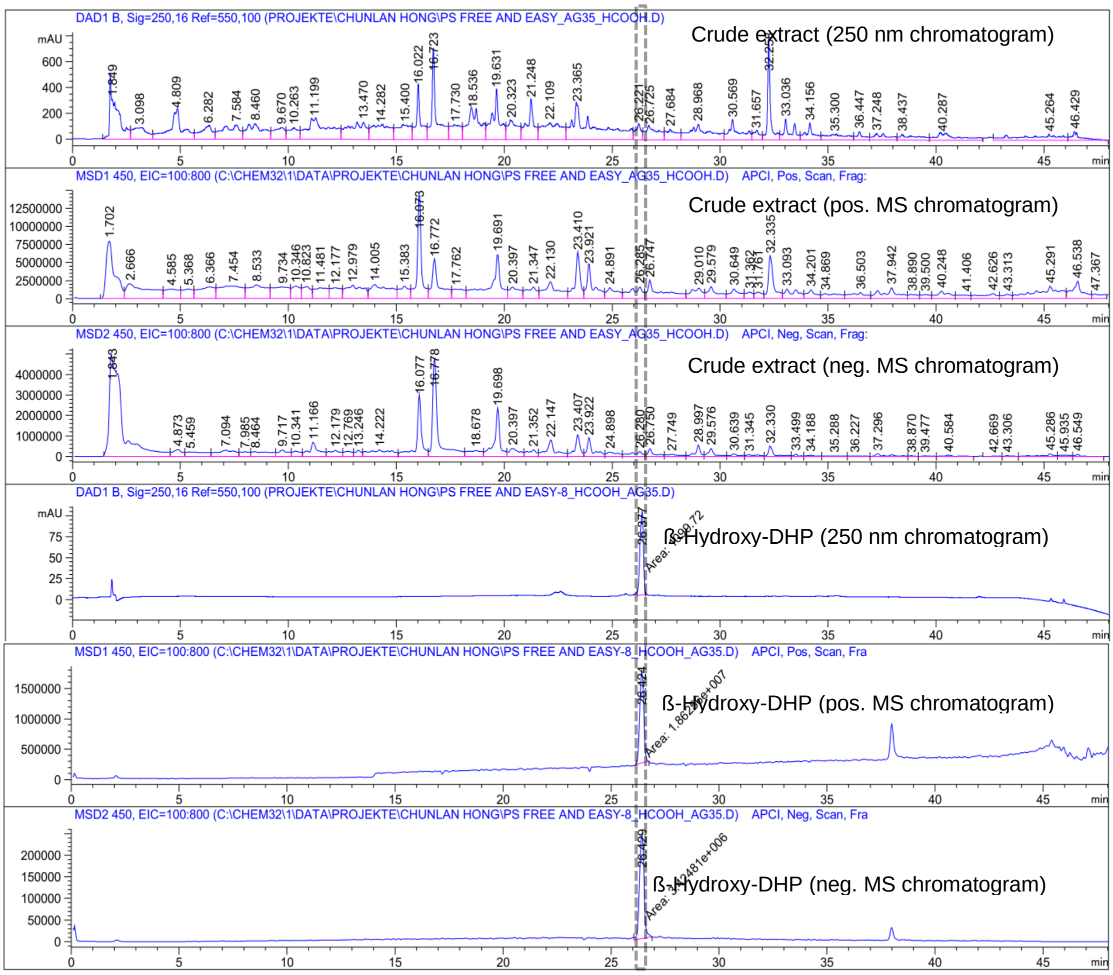

□-Hydroxy-DHP: MS-spectra (comparison of corresponding MS spectra)

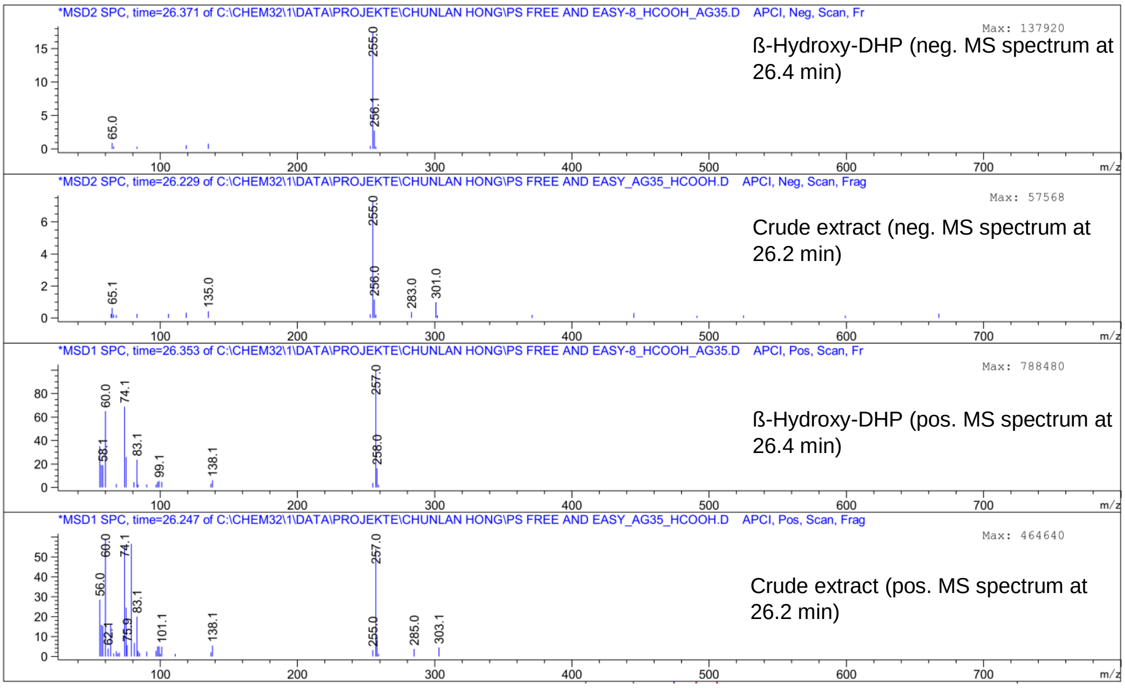

1.9. FAEW-9 Oroxyloside

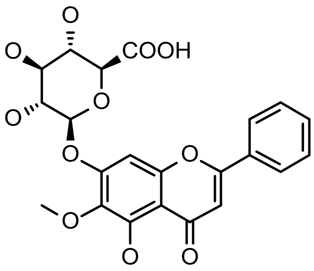

Oroxyloside
FEW-9

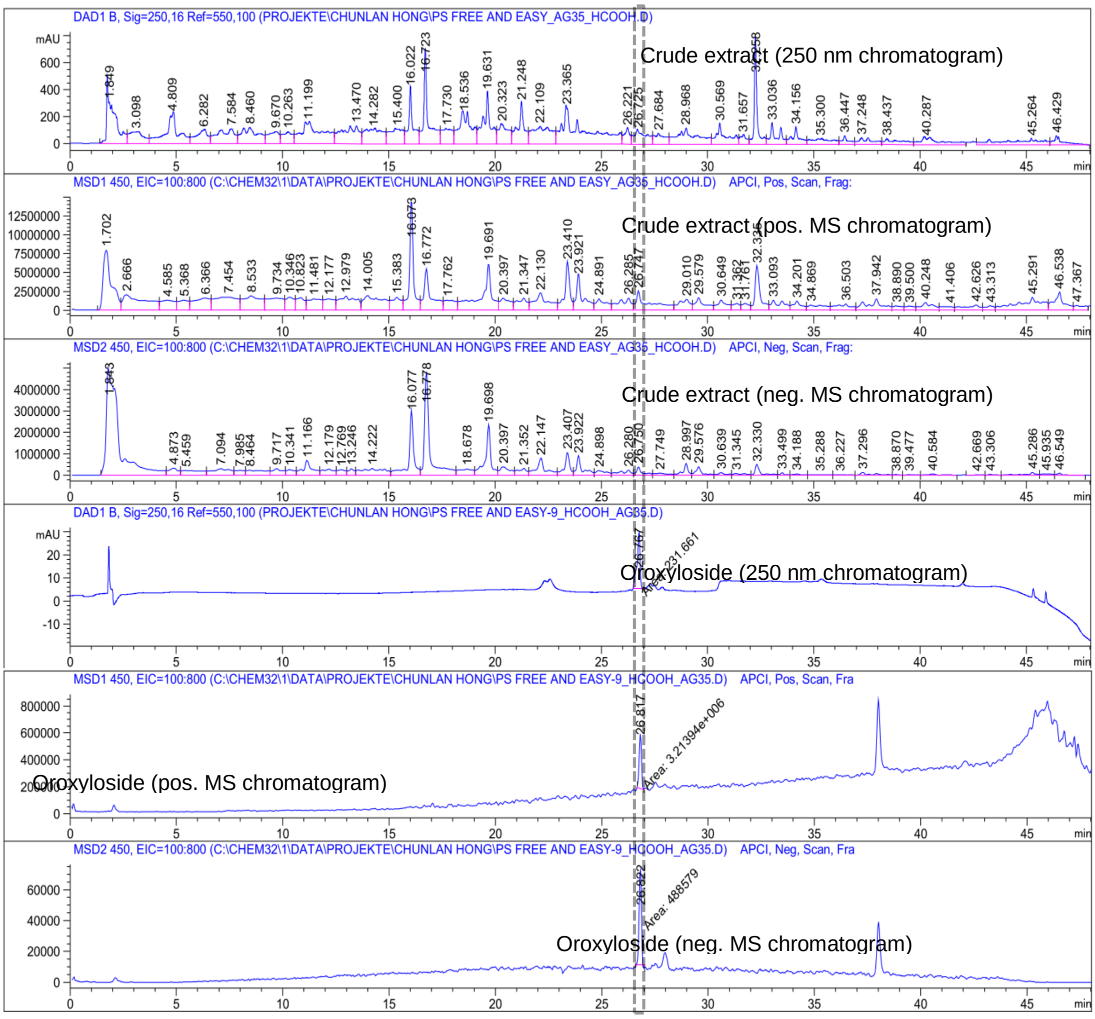

Oroxyloside: MS-spectra (comparison of corresponding MS spectra)

2 Structure identification

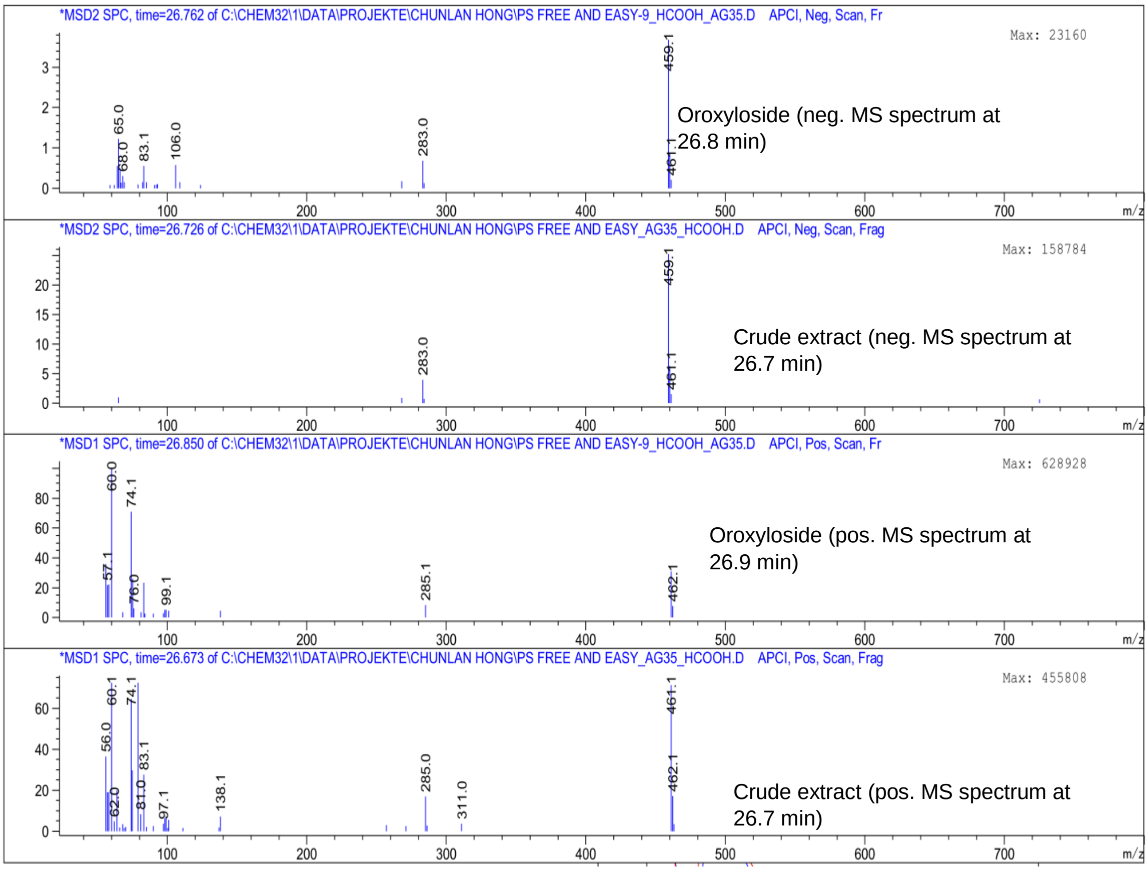

2. 1 FAEW-1

Brown oil, $[\alpha]_D^{29}$= –7.3 (c = 0.07, MeOH), ESI-MS m/z = 481.2 $[M+H]^+$, HR-ESI-MS m/z = 503.1529 calculated for $[C_{23}H_{28}O_{11}+Na]^+$; found: 503.1546.

^{1}H NMR, COSY (600 MHz, Methanol-d_4) δ 8.11 – 8.07 (m, 2H, H-2‘‘/H-6‘‘), 7.64 – 7.60 (m, 1H, H-4‘‘), 7.52 – 7.48 (m, 2H, H-3‘‘/H-5‘‘), 4.80 (d, J = 12.2 Hz, 1H, H-8), 4.68 (d, J = 12.2 Hz, 1H, H-8), 4.53 (d, J = 7.6 Hz, 1H, H-1‘), 4.27 (dd, J = 6.6, 4.9 Hz, 1H, H-4), 3.85 (dd, J = 11.8, 1.5 Hz, 1H, H-6‘), 3.61 – 3.58 (m, 1H, H-6‘), 3.27 – 3.24 (m, 1H, H-3‘), 3.24 – 3.19 (m, 3H, H-2‘, H-4‘, H-5‘), 2.92 (dd, J = 7.8, 4.9 Hz, 1H, H-5), 2.80 (dd, J = 11.0, 7.8 Hz, 1H, H-7), 2.41 (dd, J = 15.4, 6.6 Hz, 1H, H-3), 2.05 (d, J = 11.0 Hz, 1H, H-7), 2.00 (dd, J = 15.4, 1.5 Hz, 1H, H-3), 1.52 (s, 3H, C-2-*Me*).

^{13}C NMR, HSQC, HMBC (151 MHz, MeOD) δ 178.0 (C-9), 167.9 (C-1‘‘-*C*O), 134.4 (C-4’’), 131.2 (C-1’’), 130.8 (C-2’’/C-6’’), 129.7 (C-3’’/C-5’’), 100.1 (C-1’), 93.5 (C-2), 86.9 (C-1), 78.2 (C-5’), 78.0 (C-3’), 74.8 (C-2’), 71.6 (C-4’), 68.4 (C-4), 62.8 (C-6’), 62.0 (C-8), 56.9 (C-6), 41.7 (C-5), 41.6 (C-3), 28.5 (C-7), 20.6 (C-2-*Me*).

2.2. FAEW-2

Brown oil, $[\alpha]_D^{29}$= –23.2 (*c* = 0.17, MeOH), ESI-MS *m/z* = 503.2 $[M+Na]^+$, HR-ESI-MS *m/z* = 503.1529 calculated for $[C_{23}H_{28}O_{11}+Na]^+$; found: 503.1523

^1H NMR, COSY (600 MHz, Methanol-d_4) δ 8.09 – 8.04 (m, 2H, H-2‘‘/H-6‘‘), 7.66 – 7.60 (m, 1H, H-4‘‘), 7.52 – 7.48 (m, H-3‘‘/H-5‘‘), 5.42 (s, 1H, H-9), 4.78 – 4.72 (m, 2H, H-8), 4.53 (d, *J* = 7.7 Hz, 1H, H-1‘), 3.85 (dd, *J* = 11.9, 1.5 Hz, 1H, H-6‘), 3.63 – 3.58 (m, 1H, H-6‘), 3.31 (m, 1H, H-3‘), 3.25 – 3.20 (m, 3H, H-2‘, H-4‘, H-5‘), 2.62 – 2.57 (m, 1H, H-5), 2.50 (dd, *J* = 11.0, 6.9 Hz, 1H, H-7), 2.20 (d, *J* = 12.6 Hz, 1H, H-3), 1.96 (d, *J* = 11.0 Hz, 1H, H-7), 1.81 (d, *J* = 12.6, 1H, H-3), 1.37 (s, 3H, C-2-*Me*).

^{13}C NMR, HSQC, HMBC (151 MHz, MeOD) δ 167.95 (C-1‘‘-*C*O), 134.43 (C-4’’), 131.21 (C-1’’), 130.68 (C-2’’/C-6’’), 129.64)C-3’’/C-5’’), 106.40 (C-4), 102.30 (C-9), 100.19 (C-1’), 89.33 (C-1), 87.25 (C-2), 78.05 (C-3’), 77.98 (C-5’), 75.01 (C-2’), 72.25 (C-6), 71.74 (C-4’), 62.87 (C-6’), 61.70 (C-8), 44.53 (C-3), 43.95 (C-5), 23.40 (C-7), 19.61 (C-2-*Me*).

2.3. FAEW-3

Yellow oil, $[\alpha]_D^{29}$= –41.8 (*c* = 0.10, MeOH), ESI-MS *m/z* = 441.1 $[M+Na]^+$, HR-ESI-MS *m/z* = 441.1161 calculated for $[C_{21}H_{22}O_9+Na]^+$; found: 441.1169

^1H NMR, COSY (600 MHz, Methanol-d_4) δ 7.74 (d, *J* = 8.7 Hz, 1H, H-5), 7.48 – 7.39 (m, 2H, H-2‘/H-6‘), 7.16 – 7.13 (m, 2H, H-3‘/H-5‘), 6.51 (dd, *J* = 8.7, 2.3 Hz, 1H, H-6), 6.37 (d, *J* = 2.3 Hz, 1H, H-8), 5.49 – 5.43 (m, 1H, H-2), 4.95 – 4.93 (m, 1H, H-1‘‘), 3.98 – 3.91 (m, 1H, H-6‘‘), 3.70 (dd, *J* = 12.1, 5.8 Hz, 1H, H-6‘‘), 3.49 – 3.43 (m, 3H, H-3‘‘, H-2‘‘, H-5‘‘), 3.41 –

3.37 (m, 1H, H-4‘‘), 3.05 (dd, *J* = 16.9, 12.9 Hz, 1H, H-3), 2.74 (dd, *J* = 16.9, 3.0 Hz, 1H, H-3).

^{13}C NMR, HSQC, HMBC (151 MHz, MeOD) δ 193.19 (C-4), 166.82 (C-7), 165.42 (C-8a), 159.25 (C-4‘), 134.44 (C-1‘), 129.87 (C-5), 128.78 (C-2‘/C-6‘), 117.78, 117.76 (C-3‘/C-5‘), 115.00 (C-4a), 111.80 (C-6), 103.81 (C-8), 102.17 (C-1‘‘), 80.74 (C-2), 78.19 (C-5‘‘), 77.97 (C-3‘‘), 74.89 (C-2‘‘), 71.35 (C-4‘‘), 62.48 (C-6‘‘), 45.00 (C-3).

2.4. FAEW-4

Yellow oil, $[\alpha]_D^{29}$= –46.0 (*c* = 0.05, H_2O), ESI-MS *m/z* = 447.1 $[M+H]^+$, HR-ESI-MS *m/z* = 469.0747 calculated for $[C_{21}H_{18}O_{11}+Na]^+$; found: 469.0744

^{1}H NMR, COSY (600 MHz, Deuterium Oxide) δ 7.86 (br, 1H, H-2’/H-6’), 7.53 (br, 1H, H-4’), 7.47 (br, 2H, H-3’/H-5’), 6.90 (s, 1H, H-8), 6.69 (s, 1H, H-3), 5.19 (d, *J* = 7.7 Hz, 1H, H-1’’), 3.96 (d, *J* = 9.5 Hz, 1H, H-5’’), 3.72 (t, *J* = 8.5 Hz, 1H, H-2’’), 3.69 – 3.65 (m, 1H, H-3’’), 3.62 (t, J = 9.5 Hz, H-4’’).

^{13}C NMR, HSQC, HMBC (151 MHz, D_2O) δ 175.18 (COOH), 150.26 (C-7 or 8a), 132.08 (C-4‘) , 128.94 (C-3‘/C-5‘), 126.25 (C-2‘/C-6‘), 106.55 (C-4a), 104.17 (C-3), 99.73 (C-1‘‘), 94.29 (C-8), 76.48 (C-5‘‘), 74.94 (C-3‘‘), 72.39 (C-2‘‘), 71.59 (C-4‘‘).

2.5. FAEW-5

Brown oil, $[\alpha]_D^{29}$= +17.4 (*c* = 0.13, MeOH), ESI-MS *m/z* = 941.1 $[M+H]^+$, HR-ESI-MS *m/z* = 963.1079 calculated for $[C_{41}H_{32}O_{26}+Na]^+$; found: 963.1052

^{1}H NMR, COSY (600 MHz, Methanol-d_4) δ 7.11 (s, 2H, H-2e/H-6e), 7.05 (s, 1H, H-2a/H-6a), 6.98 (s, 2H, H-2d/H-6d), 6.95 (s, 2H, H-2b/H-6b), 6.90 (s, 2H, H-2c/H-6c), 6.24 (d, *J* = 8.3 Hz, 1H, H-1), 5.91 (t, *J* = 9.7 Hz, 1H, H-3), 5.64 – 5.57 (m, 2H, H-4, H-2), 4.51 (dd, *J* = 12.3, 1.9 Hz, 1H, H-6), 4.43 – 4.36 (m, 2H, H-5, H-6).

^{13}C NMR, HSQC, HMBC (151 MHz, MeOD) δ 167.91 (C-1e-*C*O), 167.28 (C-1c-*C*O), 167.01 (C-1b-*C*O), 166.91 (C-1d-*C*O), 166.20 (C-1a-*C*O), 146.54 (C-3a/C-5a), 146.47 (C-3e/C-5e),

146.43 (C-3d/C-5d), 146.37 (C-3b/C-5b), 146.27 (C-3c/C-5c), 140.75 (C-4a), 140.34 (C-4d), 140.29 (C-4b), 140.11 (C-4c), 140.00 (C-4e), 121.01 (C-1e), 120.33 (C-1c), 120.21 (C-1d), 120.17 (C-1b), 119.70 (C-1a), 110.58 (C-2a/C-6a), 110.43 (C-2d/C-6d), 110.37 (C-2b/C-6b), 110.34 (C-2c/C-6c), 110.30 (C-2e/C-6e), 93.80 (C-1), 74.40 (C-5), 74.10 (C-3), 72.17 (C-2), 69.77 (C-4), 63.10 (C-6).

2.6. FAEW-6

Brown oil, ESI-MS *m/z* = 551.3 $[M+H]^+$, HR-ESI-MS *m/z* = 573.1584 calculated for $[C_{26}H_{30}O_{13}+Na]^+$; found: 573.1595

^{1}H NMR, COSY (600 MHz, Methanol-d_4) δ 8.00 (d, *J* = 8.9 Hz, 1H, H-6‘), 7.82 (d, *J* = 15.3 Hz, 1H, H-3), 7.75 – 7.69 (m, 3H, H-2‘‘/H-6‘‘, H-2), 7.15 – 7.12 (m, 2H, H-3‘‘/H-5‘‘), 6.43 (dd, *J* = 8.9, 2.4 Hz, 1H, H-5‘), 6.30 (d, *J* = 2.4 Hz, 1H, H-3‘), 5.47 (d, *J* = 1.6 Hz, 1H, H-1^{iv}), 5.06 (d, *J* = 7.6 Hz, 1H, H-1’’’), 4.06 (d, J = 9.6 Hz, 1H, H-4^{iv}), 3.95 (d, *J* = 1.6 Hz, 1H, H-2^{iv}), 3.94 – 3.89 (m, 1H, H-6’’’), 3.81 (d, *J* = 9.6 Hz, 1H, H-4d), 3.74 – 3.69 (m, 1H, H-6’’’), 3.67 (dd, *J* = 9.0 Hz, 7.6 Hz, 1H, H-2c), 3.62 (t, *J* = 9.0 Hz, 1H, H-3c), 3.58 – 3.54 (m, 1H, H-5’’’ or H-5‘‘ Ononin), 3.54 (d, *J* = 2.1 Hz, 2H, H-5^{iv}), 3.50 – 3.46 (m, 1H, H-5’’’ or H-5‘‘ Ononin), 3.43 – 3.38 (m, 1H, H-4’’’).

^{13}C NMR, HSQC, HMBC (151 MHz, MeOD) δ 193.36 (C-1), 167.61 (C-2‘), 166.54 (C-4‘), 160.95 (C-4‘‘), 144.82 (C-3), 133.50 (C-6‘), 131.46 (C-2‘‘/C-6‘‘), 130.49 (C-1‘‘), 120.03 (C-2), 117.84 (C-3‘‘/C-5‘‘), 114.67 (C-1‘), 110.82 (C-1^{iv}), 109.20 (C-5‘), 103.79 (C-3‘), 100.43 (C-1‘‘‘), 80.76 (C-3^{iv}), 78.60 (C-3‘‘‘), 78.57 (C-2‘‘‘), 78.42 (C-5’’ Ononin or C-5‘‘‘), 78.17 (C-5’’ Ononin or C-5‘‘‘), 78.05 (C-2^{iv}), 75.47 (C-4^{iv}), 71.33 (C-4‘‘‘ or C-4‘‘ Ononin), 71.23 (C-4‘‘‘ or C-4‘‘ Ononin), 66.00 (C-5d^{iv}), 62.43 (C-6’’’).

ESI-MS *m/z* = 431.2 $[M+H]^+$, HR-ESI-MS *m/z* = 453.1161 calculated for $[C_{22}H_{22}O_9+Na]^+$; found: 453.1172

^{1}H NMR, COSY (600 MHz, Methanol-d_4) δ 8.24 (s, 1H, H-2), 8.16 (d, *J* = 8.9 Hz, 1H, H-5), 7.52 – 7.48 (m, 2H, H-2'/H-6'), 7.26 (d, *J* = 2.3 Hz, 1H, H-8), 7.23 (dd, *J* = 8.9, 2.3 Hz, 1H, H-6), 7.02 – 6.98 (m, 2H, H-3'/H-5'), 5.13 – 5.10 (m, 1H, H-1''), 3.94 – 3.89 (m, 1H, H-6''), 3.83 (s, 3H, C-4'-OMe), 3.74 – 3.69 (m, 1H, H-6''), 3.58 – 3.54 (m, 1H, H-5'' or H-5''' FEW-6a), 3.53 – 3.51 (m, 2H, H-2'', H-3''), 3.50 – 3.46 (m, 1H, H-5'' or H-5''' FEW-6a), 3.43 – 3.38 (m, 1H, H-4'').

^{13}C NMR, HSQC, HMBC (151 MHz, MeOD) δ 177.97 (C-4), 163.53 (C-7), 161.20 (C-4'), 159.25 (C-8a), 155.26 (C-2), 131.37 (C-2'/C-6'), 128.30 (C-5), 125.97 (C-3), 125.29 (C-1'), 120.21 (C-4a), 117.08 (C-6), 114.86 (C-3'/C-5'), 104.94 (C-8), 101.79 (C-1''), 78.42 (C-5'' or C-5''' FEW-6a), 78.17 (C-5'' or C-5''' FEW-6a), 77.84 (C-3''), 74.73 (C-2''), 71.33 (C-4'' or C-4''' FEW-6a), 71.23 (C-4'' or C-4''' FEW-6a), 62.43 (C-6''), 55.73 (C-4'-*Me*).

2.7. FAEW-7

Brown oil, $[\alpha]_D^{29}$= –35.4 (*c* = 0.16, MeOH), ESI-MS *m/z* = 419.2 $[M+H]^+$, HR-ESI-MS *m/z* = 441.1161 calculated for $[C_{21}H_{22}O_9+Na]^+$; found: 441.1167

^{1}H NMR, COSY (600 MHz, Methanol-d_4) δ 8.00 (d, *J* = 9.0 Hz, 1H, H-6'), 7.82 (d, *J* = 15.4 Hz, 1H, H-3), 7.75 – 7.69 (m, 3H, H-2''/H-6'', H-2), 7.19 – 7.14 (m, 2H, H-3''/H-5''), 6.43 (dd, *J* = 9.0, 2.4 Hz, 1H, H-5'), 6.30 (d, *J* = 2.4 Hz, 1H, H-3'), 5.00 (dd, *J* = 5.4, 2.2 Hz, 1H, H-1'''), 3.91 (dd, *J* = 12.1, 2.3 Hz, 1H, H-6'''), 3.71 (dd, *J* = 12.1, 5.7 Hz, 1H, H-6'''), 3.51 – 3.46 (m, 3H, H-2''', H-3''', H-5'''), 3.43 – 3.37 (m, 1H, H-4''').

^{13}C NMR, HSQC, HMBC (151 MHz, MeOD) δ 193.37 (C-1), 167.62 (C-2'), 166.54 (C-4'), 161.08 (C-4''), 144.83 (C-3), 133.50 (C-6'), 131.42 (C-2''/C-6''), 130.53 (C-1''), 120.03 (C-2), 117.99 (C-3''/C-5''), 114.67 (C-1'), 109.20 (C-5'), 103.78 (C-3'), 101.80 (C-1'''), 78.28 (C-5'''), 77.95 (C-3'''), 74.83 (C-2'''), 71.29 (C-4'''), 62.46 (C-6''').

2.8. FAEW-8

Yellow oil, $[\alpha]_D^{29}$= –8.9 (*c* = 0.06, MeOH), ESI-MS *m/z* = 256.1 $[M-H_2O +H]^+$, HR-ESI-MS *m/z* = 279.0633 calculated for $[C_{15}H_{14}O_5–H_2O +Na]^+$; found: 279.0645

^{1}H NMR, COSY (600 MHz, Methanol-d_4) δ 7.74 (d, *J* = 8.7 Hz, 1H, H-6), 7.35 – 7.31 (m, 2H, H-2‘/H-6‘), 6.86 – 6.79 (m, 2H, H-3‘/H-5‘), 6.50 (dd, *J* = 8.7, 2.3 Hz, 1H, H-5), 6.36 (d, *J* = 2.3 Hz, 1H, H-3), 5.39 (dd, *J* = 13.2, 2.9 Hz, 1H, H-β), 3.06 (dd, *J* = 16.9, 13.1 Hz, 1H, H-α), 2.70 (dd, *J* = 16.9, 2.9 Hz, 1H, H-α).

^{13}C NMR, HSQC, HMBC (151 MHz, MeOD) δ 193.54 (*C*=O), 166.79 (C-2), 165.58 (C-4), 158.99 (C-4’), 131.34 (C-1’), 129.85 (C-6), 129.02 (C-2’/C-6’), 116.28 (C-3’/C-5‘), 114.97 (C-1), 111.72 (C-5), 103.79 (C-3), 81.07 (Cβ), 44.97 (Cα).

2.9. FAEW-9

Brown oil, $[\alpha]_D^{29}$= –10.0 (*c* = 0.05, MeOH), ESI-MS *m/z* = 461.2 $[M+H]^+$, HR-ESI-MS *m/z* = 483.0903 calculated for $[C_{22}H_{20}O_{11}+Na]^+$; found: 483.0923

^{1}H NMR, COSY (600 MHz, Methanol-d_4) δ 8.07 (d, *J* = 7.4 Hz, 2H, H-2‘/H-6‘), 7.64 – 7.57 (m, 2H, H-3‘/H-5‘), 6.85 (s, 1H, H-3), 6.67 (s, 1H, H-8), 5.21 (d, *J* = 7.3 Hz, 1H, H-1‘‘), 4.06 (d, *J* = 7.8 Hz, H-5‘‘), 3.99 (s, 3H, C-6-O*Me*), 3.67 – 3.59 (m, 2H, H-2‘‘, H-4‘‘), 3.58 – 3.52 (m, 1H, H-3‘‘).

^{13}C NMR, HSQC, HMBC (151 MHz, MeOD) δ 184.32 (C-4), (C6’’ not observed), 165.97 (C-2), 158.11 (C-7), 157.73 (C-5), 151.14 (C-8a), 133.34 (C-4’), 132.48 (C-1’), 131.27 (C-6), 130.35 (C-3’/C-5’), 127.57 (C-2’/C-6’), 107.23 (C-4a), 106.20 (C-3), 101.84 (C-1’’), 100.34 (C-8), 77.40 (C-3’’), 76.67 (C-5’’), 74.51 (C-2’’), 72.93 (C-4’’), 62.50 (C-6-O*Me*).

Methods for the image of blotting

- Protocols: After the protein was transferred from the get to the membrane, Ponceau S was used to check whether transfer was successful, with the help of marker, we can see the bands of the proteins very clear, then the membranes were crippled to different parts, as NRF2 (97-100), KEAP1 (60-64), HO-1 (28), β-actin (43), LaminA/C (Lamin A: 74, Lamin C: 63) and TBP (38) were located in different parts of the membranes. Each protein was measured separately after incubating the substrate in dark for 1-2 min.

- Instrument: FluorChem® Q from Biozym Scientific GmbH

- Substrate: luminata™ Classico Western HRP substrate or Western Bright Chemilumineszenz Substrate Sirius

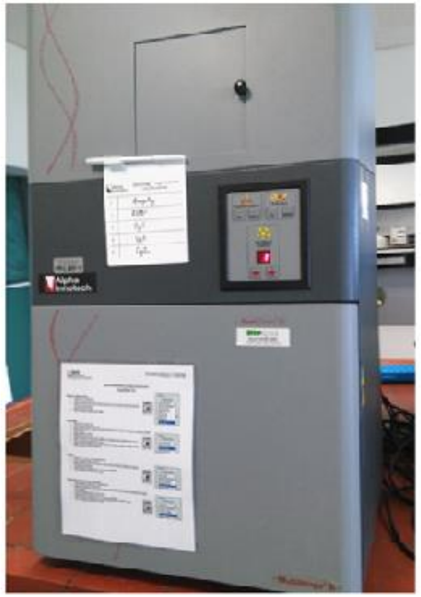

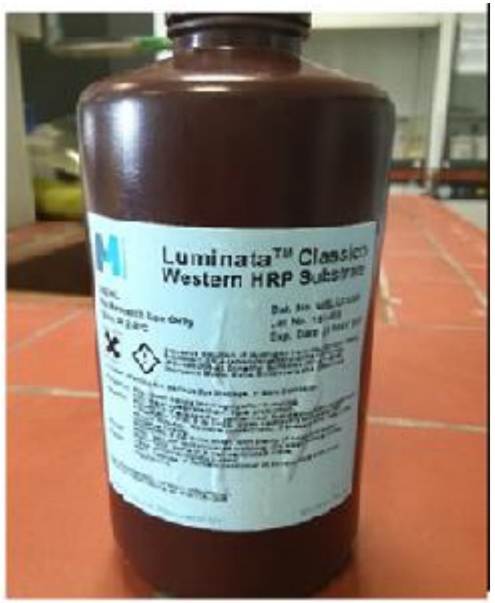

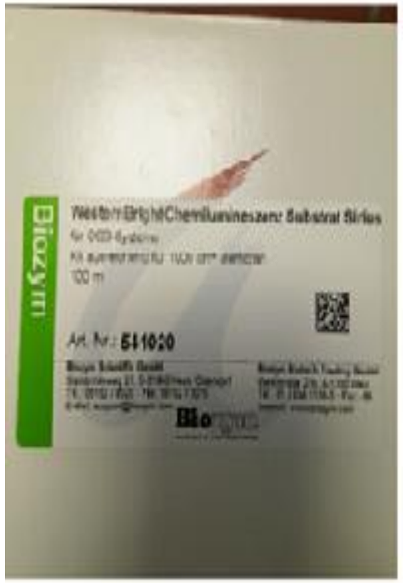

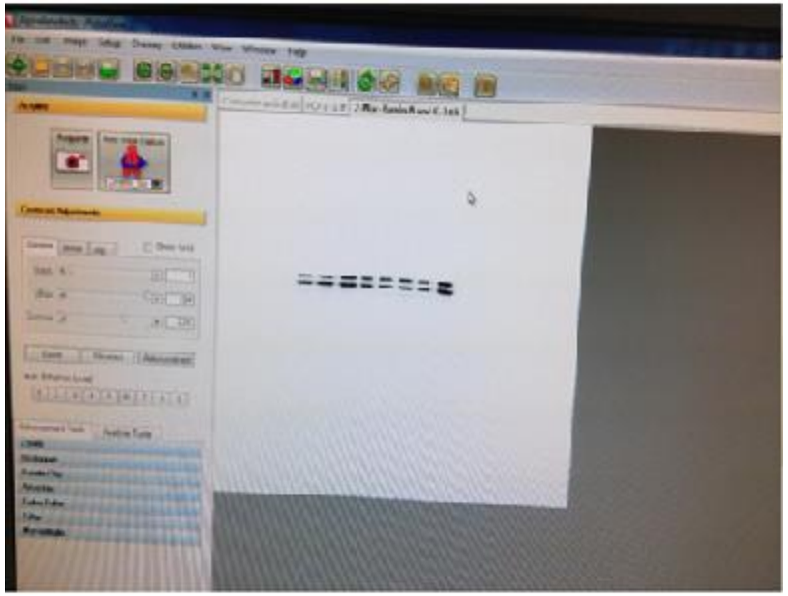

T98G cell line-24 h H_2O_2

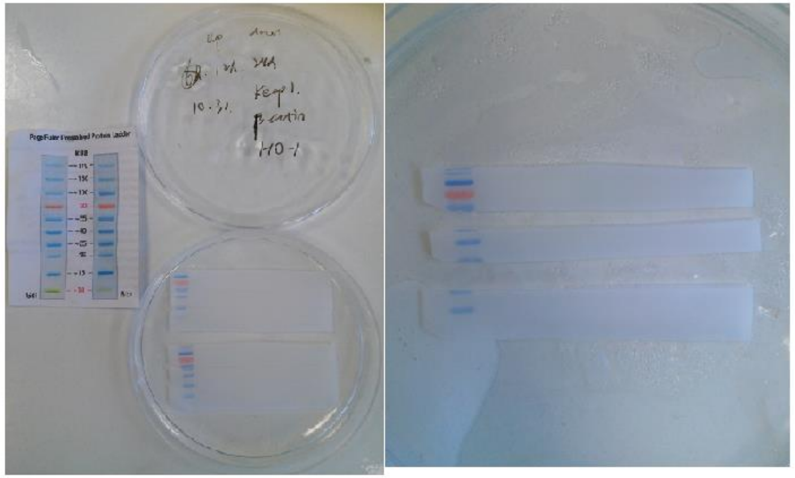

KEAP1

β-actin

HO-1

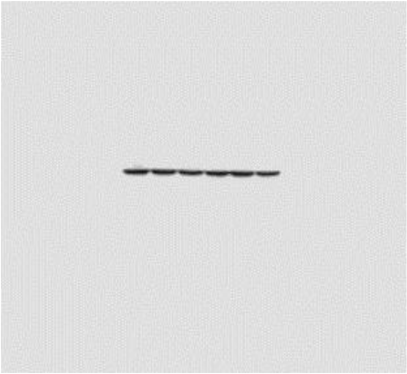

beta-actin-24h

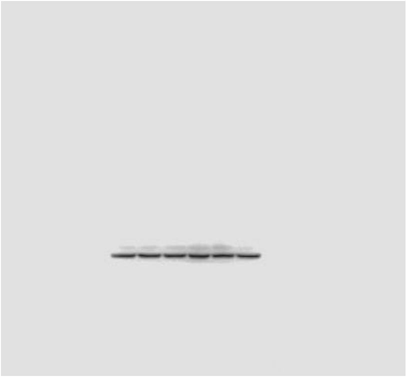

CAT-24h

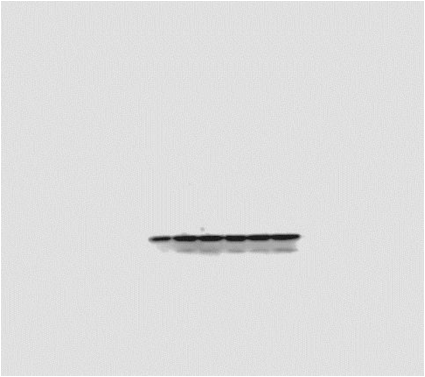

HO-1-24h

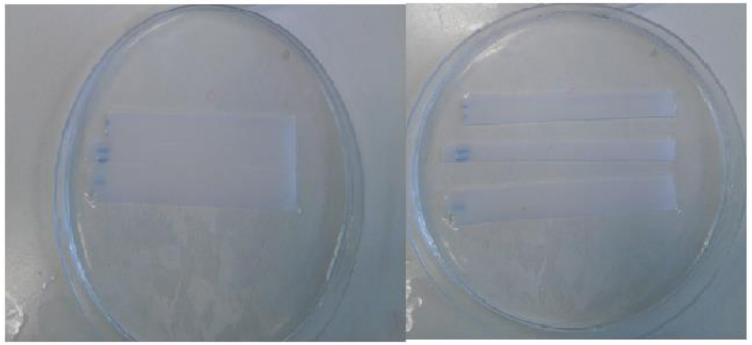

N-NRF2

Lamin A/C

TBP

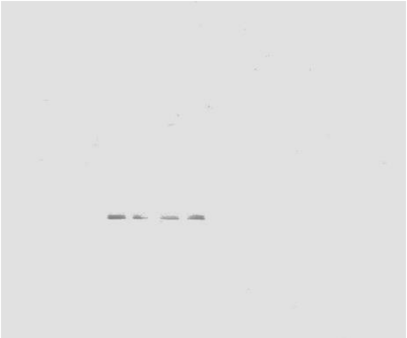

KEAP1-24h

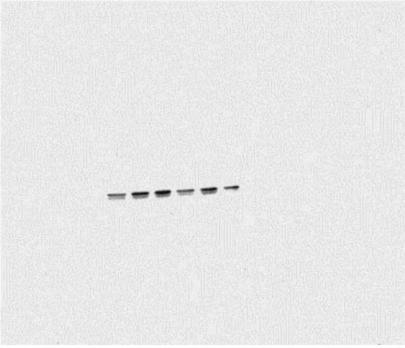

NRF2-24h

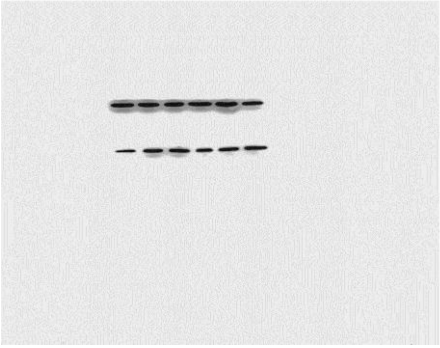

TBP-24h-up

T98G cell line-12 h H_2O_2

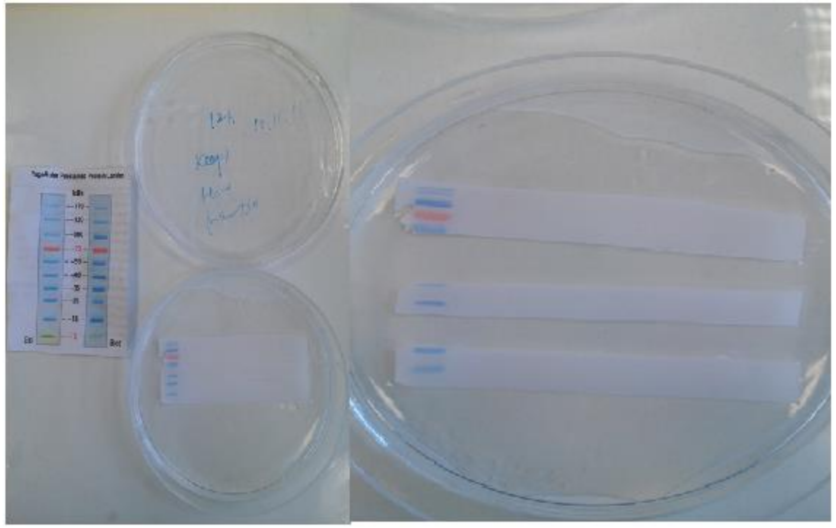

KEAP1
β-actin
HO-1

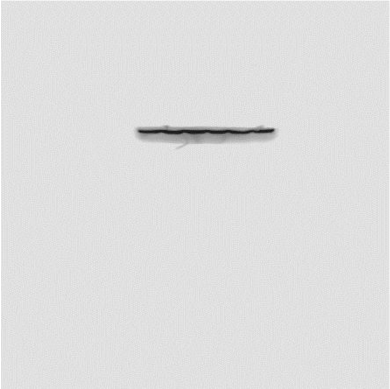

ACTIN-12h

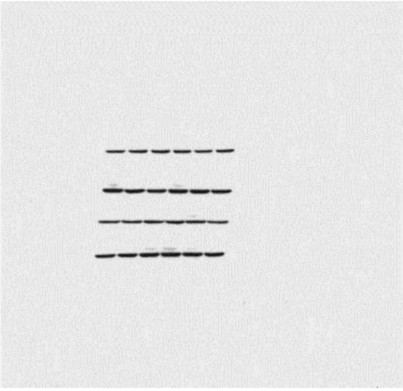

CAT-12h-the second one

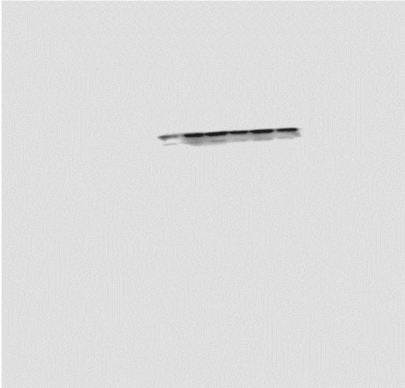

HO-1-12h

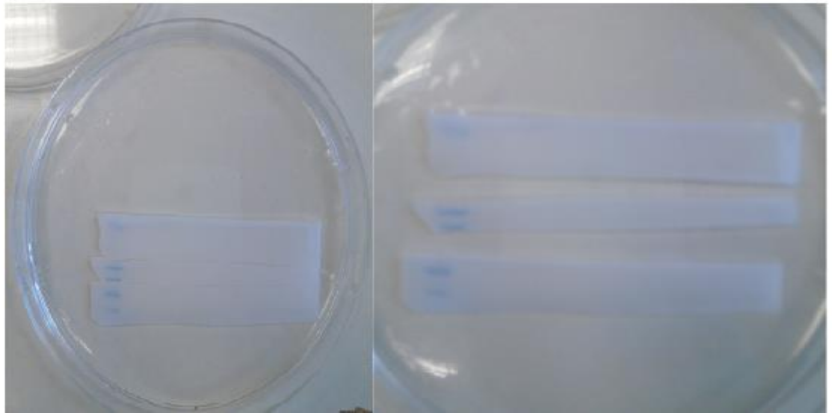

N-NRF2
Lamin A/C
TBP

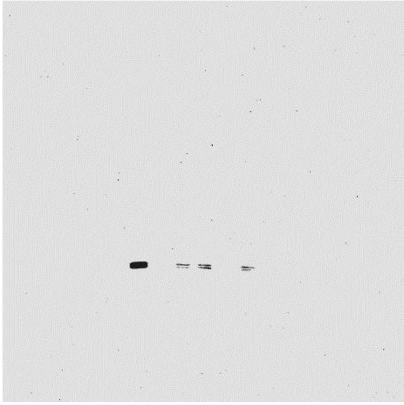

KEAP1-12h

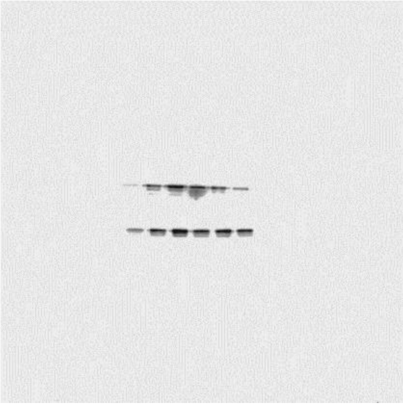

NRF2-12h-UP

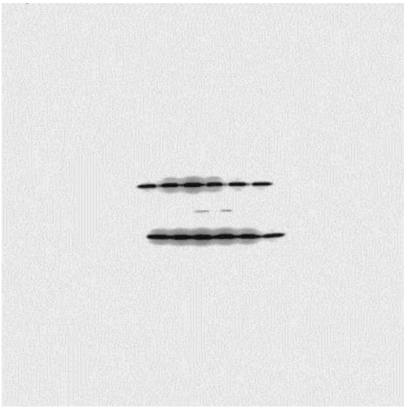

TBP-12h-UP

T98G cell line-6h H_2O_2

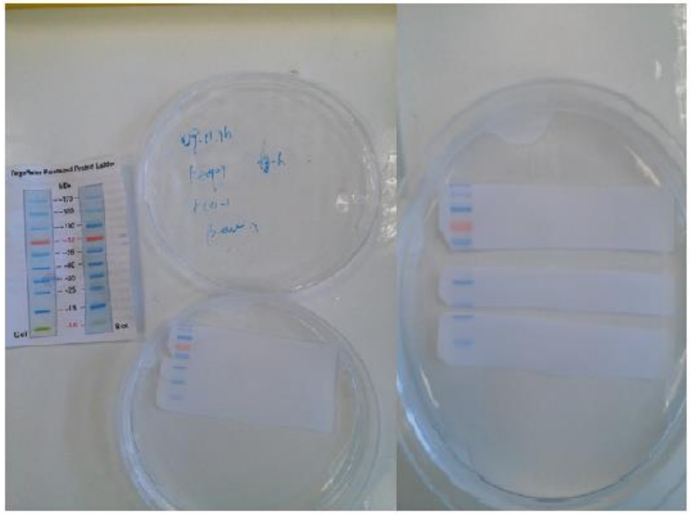

KEAP1

β-actin

HO-1

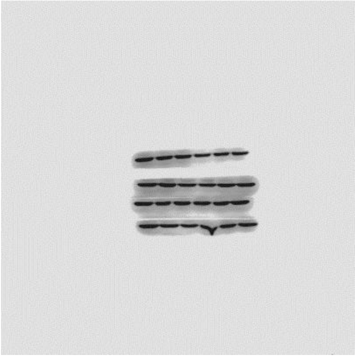

actin-6h-the third one

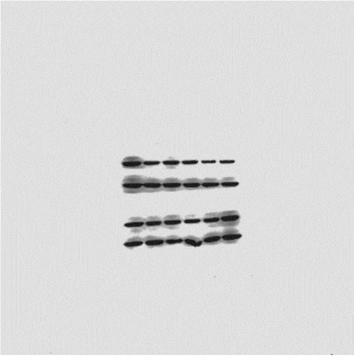

CAT-6h-the third one

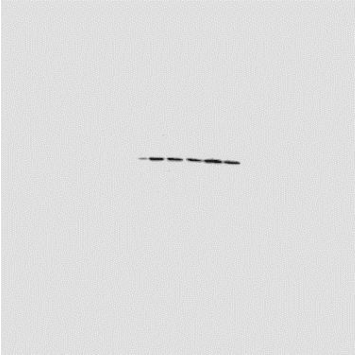

HO-1-6h

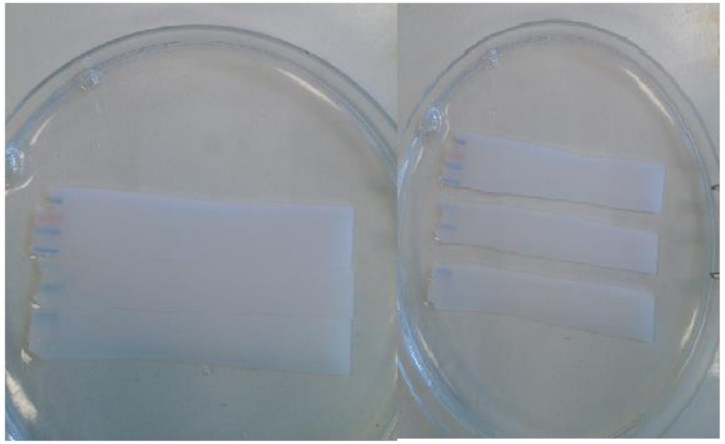

N-NRF2

TBP

Histone

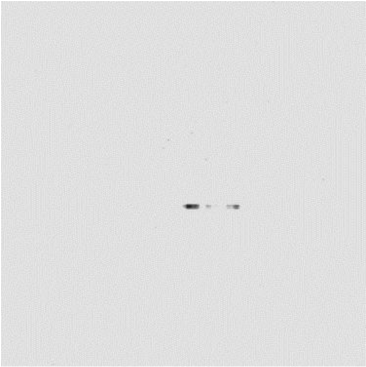

KEAP1-6h

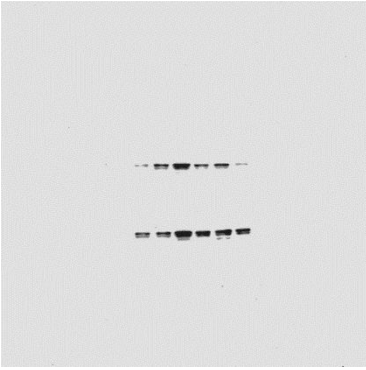

NRF2-6h-down

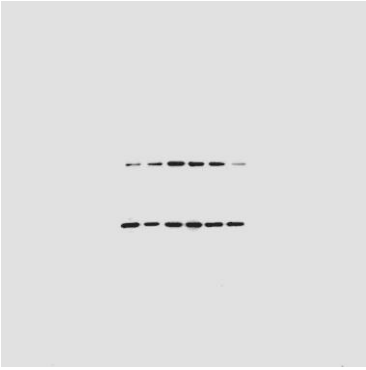

TBP-6h-down

T98G cell line-10 min H_2O_2

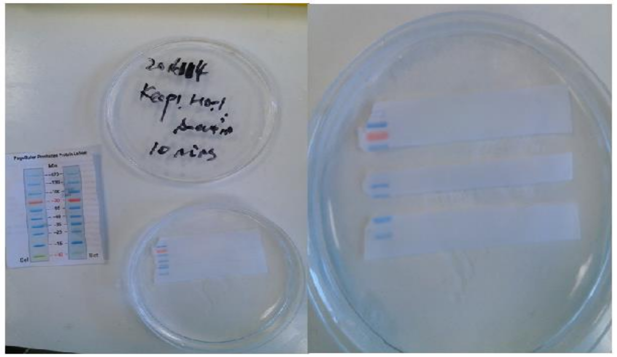

KEAP1
β-actin
HO-1

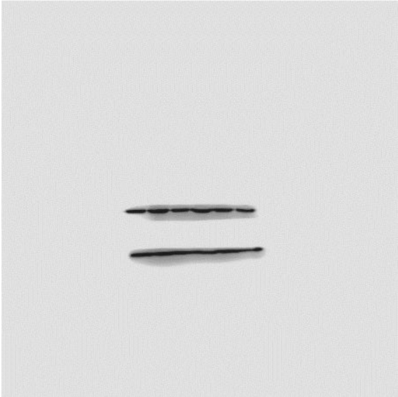

ACTIN-10 MINS-UP

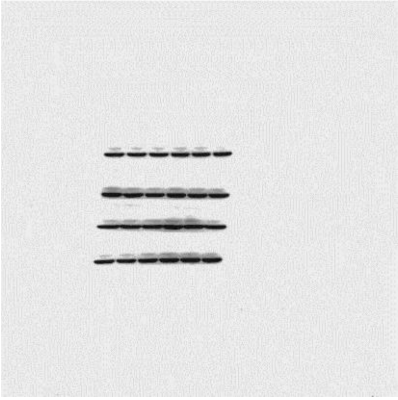

CAT-10 MINS-the last one

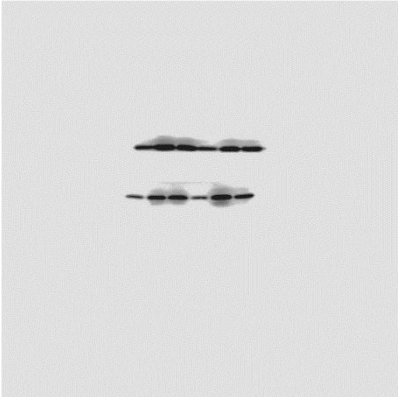

HO-1-10 MINS-UP

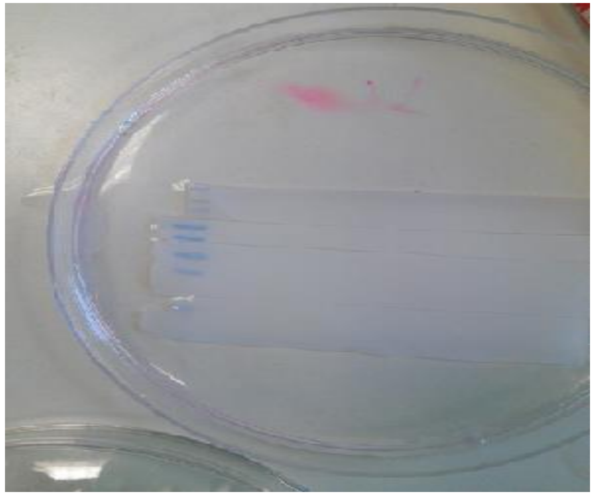

N-NRF2
Lamin A/C
TBP

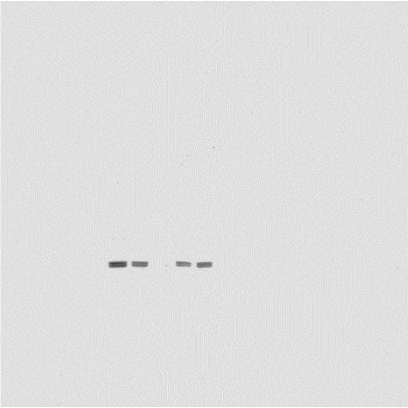

KEAP1-10 MINS

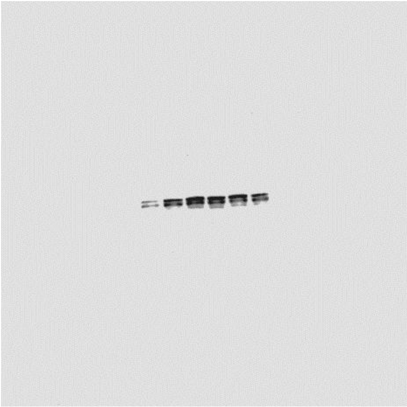

NRF2-10 MINS

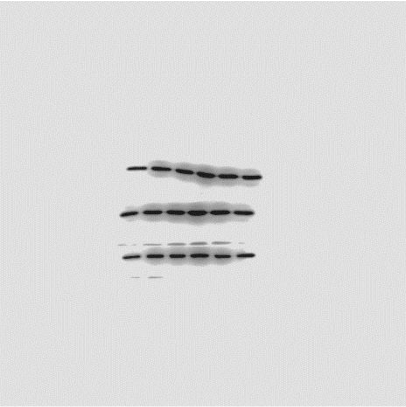

TBP-10 MINS-up

T98G cell line-12 h

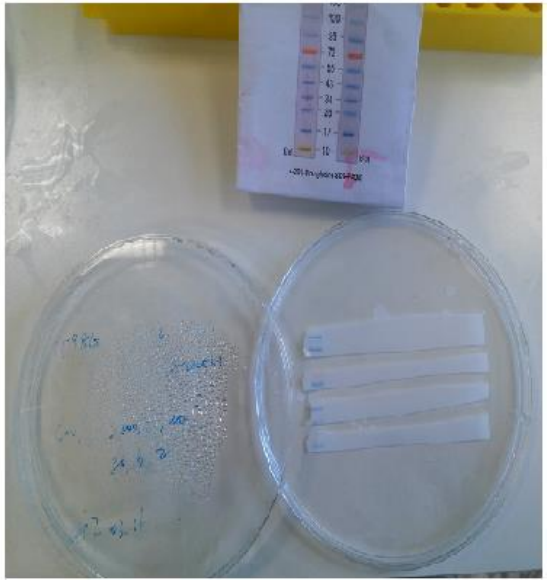

C-NRF2
KEAP1
β-actin
HO-1

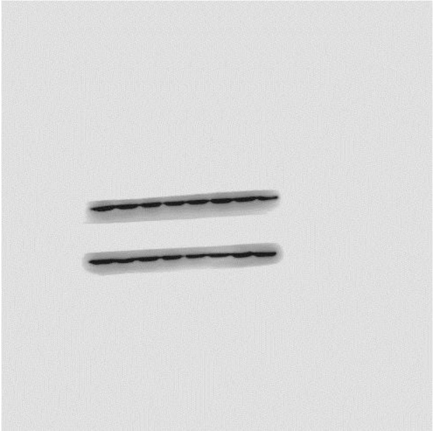

12h-T98G-actin-up

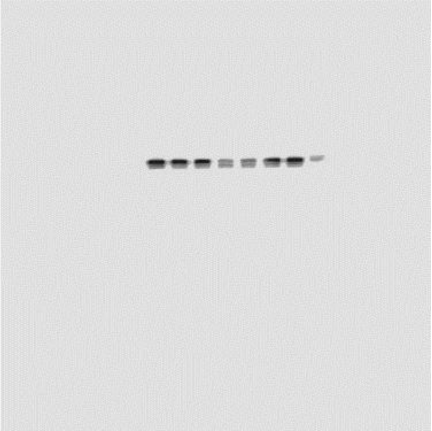

12h-T98G-C-NRF2

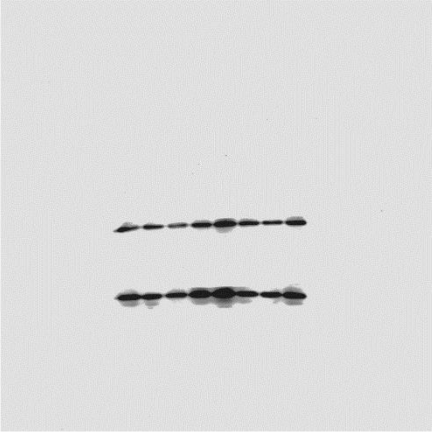

12h-T98G-HO-1-UP

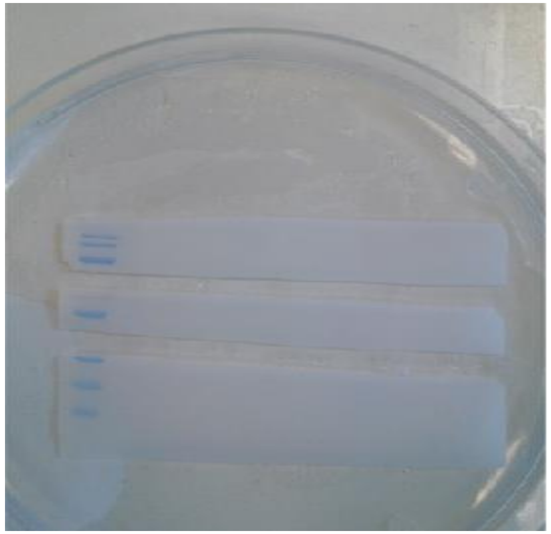

N-NRF2

Lamin A/C

TBP

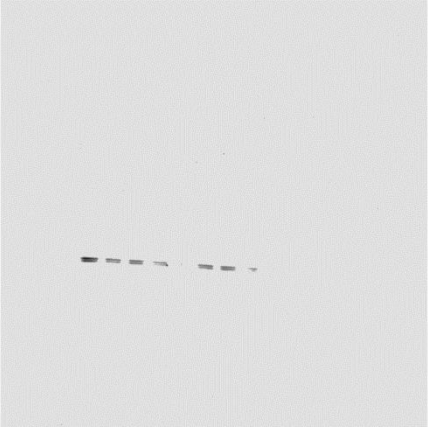

12h-T98G-KEAP1

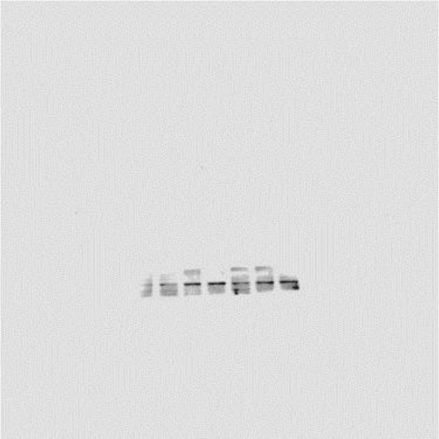

12h-T98G-N-NRF2

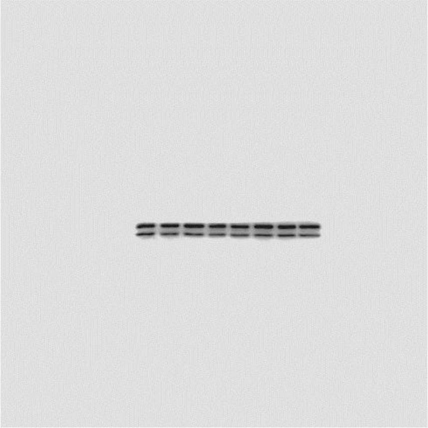

Lamin A and C-12h-T98G

SH-SY5Y cell line-12h

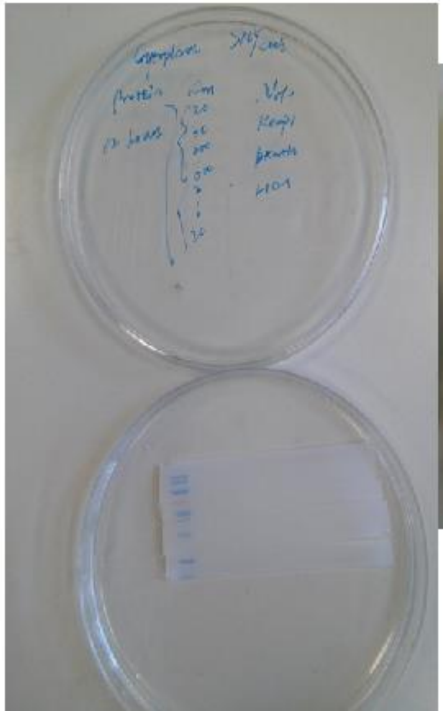

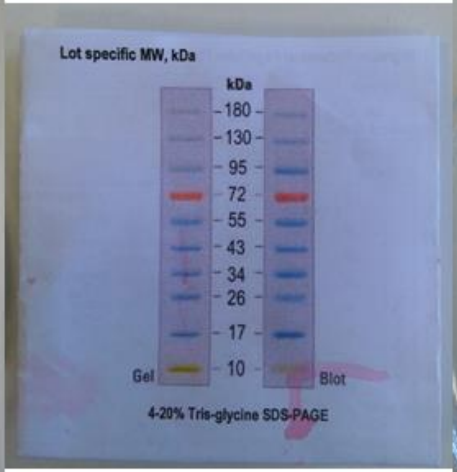

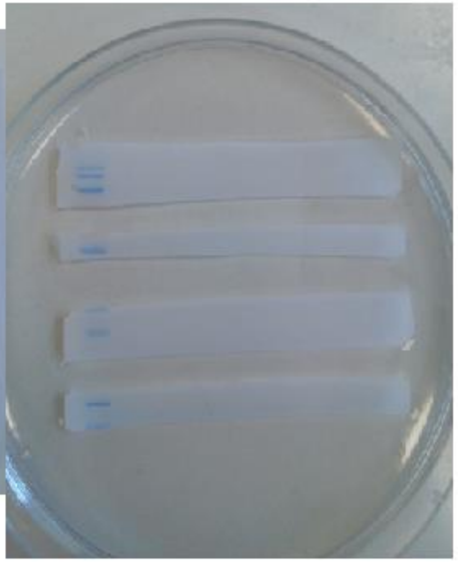

C-NRF2

KEAP1

β-actin

HO-1

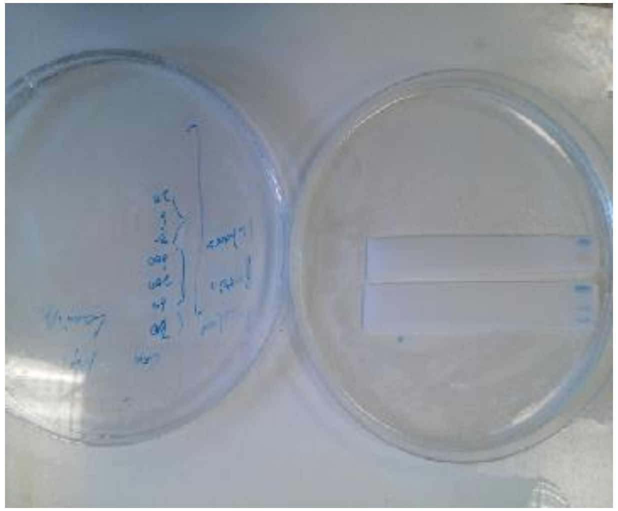

N-NRF2
Lamin A/C

SY-SH5Y cell line-12 h

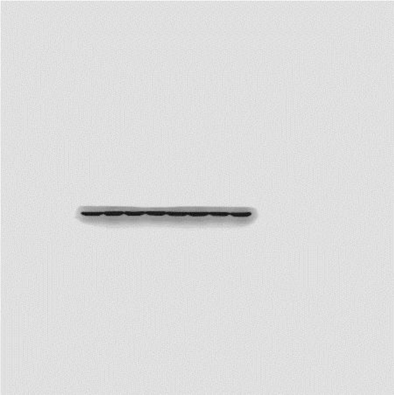
12h-SH-SY5Y-ACTIN

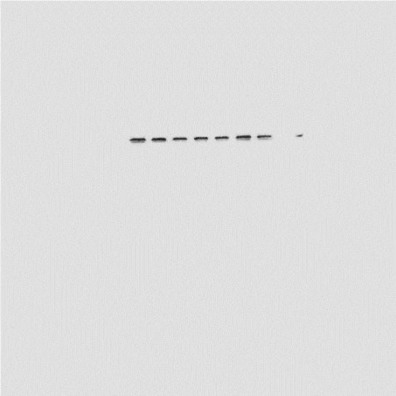
12h-SH-SY5Y-C-NRF2

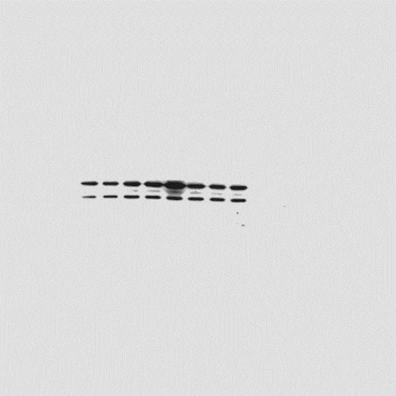
12h-SH-SY5Y-HO-1

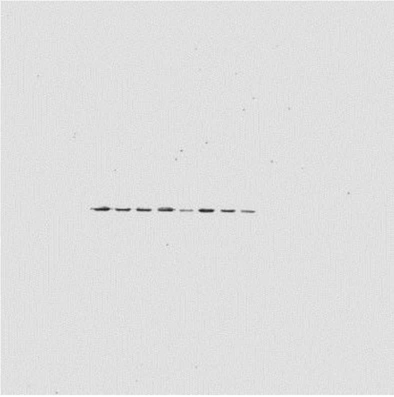
12h-SH-SY5Y-KEAP1

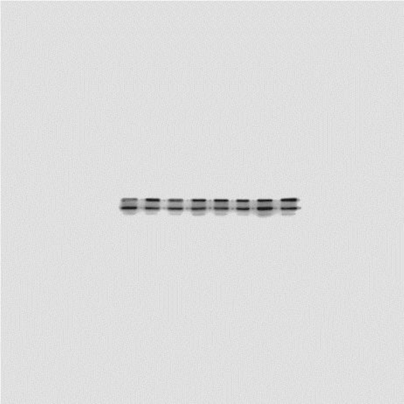
12h-SH-SY5Y-LAMIN A/C

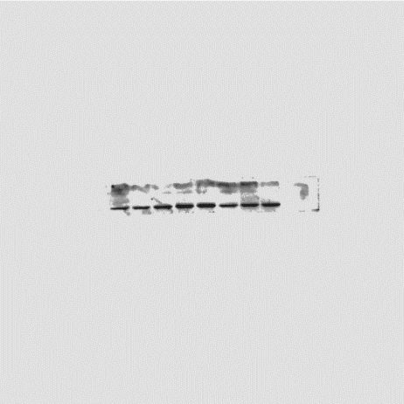
12h-SH-SY5Y-N-NRF2
